# Damage/Danger Associated Molecular Patterns (DAMPs) Modulate *Chlamydia pecorum* and *C*. *trachomatis* Serovar E Inclusion Development *In Vitro*

**DOI:** 10.1371/journal.pone.0134943

**Published:** 2015-08-06

**Authors:** Cory Ann Leonard, Robert V. Schoborg, Nicole Borel

**Affiliations:** 1 Department of Pathobiology, Institute of Veterinary Pathology, University of Zurich, Zurich, Switzerland; 2 Department of Biomedical Sciences, Center for Inflammation, Infectious Disease and Immunity, James H. Quillen College of Medicine, East Tennessee State University, Johnson City, Tennessee, United States of America; Auburn University, UNITED STATES

## Abstract

Persistence, more recently termed the chlamydial stress response, is a viable but non-infectious state constituting a divergence from the characteristic chlamydial biphasic developmental cycle. Damage/danger associated molecular patterns (DAMPs) are normal intracellular components or metabolites that, when released from cells, signal cellular damage/lysis. Purine metabolite DAMPs, including extracellular ATP and adenosine, inhibit chlamydial development in a species-specific manner. Viral co-infection has been shown to reversibly abrogate *Chlamydia* inclusion development, suggesting persistence/chlamydial stress. Because viral infection can cause host cell DAMP release, we hypothesized DAMPs may influence chlamydial development. Therefore, we examined the effect of extracellular ATP, adenosine, and cyclic AMP exposure, at 0 and 14 hours post infection, on *C*. *pecorum* and *C*. *trachomatis* serovar E development. In the absence of *de novo* host protein synthesis, exposure to DAMPs immediately post or at 14 hours post infection reduced inclusion size; however, the effect was less robust upon 14 hours post infection exposure. Additionally, upon exposure to DAMPs immediately post infection, bacteria per inclusion and subsequent infectivity were reduced in both *Chlamydia* species. These effects were reversible, and *C*. *pecorum* exhibited more pronounced recovery from DAMP exposure. Aberrant bodies, typical in virus-induced chlamydial persistence, were absent upon DAMP exposure. In the presence of de *novo host* protein synthesis, exposure to DAMPs immediately post infection reduced inclusion size, but only variably modulated chlamydial infectivity. Because chlamydial infection and other infections may increase local DAMP concentrations, DAMPs may influence *Chlamydia* infection *in vivo*, particularly in the context of poly-microbial infections.

## Introduction

The *Chlamydiaceae* are a genus of Gram-negative, obligate intracellular bacterial pathogens that cause a spectrum of diseases in both humans and agriculturally important animals. *C*. *pecorum* infection, for example, causes clinical manifestations in swine ranging from conjunctivitis to abortion [1]. *C*. *trachomatis* infection in humans can cause medically important conditions, such as trachoma, pelvic inflammatory disease, and infertility. Though chlamydial infections can cause acute diseases, they are most associated with chronic inflammation resulting in significant host tissue damage [2]. The chlamydiae also share a complex developmental cycle. The infectious form (the elementary body or EB), binds to and enters the host cell. After host cell entry, the EB transitions into the more metabolically active, replicative developmental form (the reticulate body or RB). The RB then grow and divide within a cytoplasmic, membrane-bound inclusion. After several rounds of division, RB convert back into infectious EB, which are released from the host cell [3]. A third stage, historically termed persistence or more recently the chlamydial stress response, is defined as a developmental stage in which the organisms are viable but non-infectious. Persistent/stressed RB are enlarged, irregularly shaped and non-dividing; these altered developmental forms are sometimes called aberrant bodies (AB). The persistence/stress response is reversible—when the stressor is removed, the chlamydiae resume normal, productive development. A variety of stressors induce chlamydial persistence/stress in culture: these include IFN-γ exposure; glucose, iron and amino acid deprivation; and host cell co-infection with herpes simplex virus type 2 [4], Human Herpes Virus 6 [5], and the coronavirus porcine epidemic diarrhea virus (PEDV) [6,7].

Both pathogen infection and sterile trauma can promote release of damage/danger associated molecular patterns (DAMPs) from mammalian cells/tissues. Typically, DAMPs are normal intracellular components or metabolites, the release of which into the extracellular milieu indicates cellular damage or lysis. Once in the extracellular environment, DAMPs bind signaling receptors on mammalian cells and initiate specific responses that promote local inflammation and wound healing. Known DAMPs include heat shock proteins, high mobility group box 1 protein (HMGB1), uric acid, and purine metabolites (reviewed in [8]) [9]. Extracellular ATP (ATP_e_) and adenosine (ADO) are two well studied DAMPs, the binding of which to purinergic receptors initiates components of the mammalian defense against intracellular pathogens (reviewed in [10]). Purinergic receptor activation is known to limit chlamydial development in a host cell- and chlamydial species-specific manner. For example, exposure of *C*. *caviae*-infected mouse J774 macrophages to ATP_e_ reduces infectious titer by >50% [11]. ATP_e_-exposure also restricts *C*. *muridarum* development in J774 cells by promoting inclusion-lysosomal fusion. However, ATP_e_-exposure does not kill chlamydiae in peritoneal macrophages elicited from P2X_7_ receptor knock-out mice, demonstrating a requirement for this receptor. ATP_e_ reduces production of *C*. *muridarum* EB in J774 cells [12] and a similar reduction is observed in HeLa cells [13]. Furthermore, *C*. *muridarum* infectious burden in P2X_7_ receptor-deficient mice is 10 times higher than that of their wild-type counterparts [13]. These data suggest that ATP_e_ may play a significant anti-chlamydial role *in vivo*, as well as *in vitro*. Extracellular ADO-exposure also reduces infectious EB production by both *C*. *trachomatis* L2 and serovar D in HeLa cells, primarily by signaling through the A2b receptor. Though recovery of serovar D was not tested, L2 recovered infectivity post-termination of ADO-exposure. Thus, the authors concluded that ADO-exposure induces the *C*. *trachomatis* L2 persistence/stress response [14].

As stated previously, host cell co-infection with PEDV abrogates productive development of both *C*. *pecorum* and *C*. *abortus* [6]. Furthermore, this effect is reversible and results in AB formation, suggesting that PEDV co-infection induces the chlamydial persistence/stress response. Neither UV inactivation of the virus nor inhibition of viral protein synthesis with cycloheximide ablates this response [7], suggesting that either a virion component (such as an envelope glycoprotein or genomic RNA) or a pre-synthesized host cellular component (such as a cytokine or DAMP) mediates this effect. Viruses induce DAMP release from infected host cells and are, in turn, affected by DAMP-induced responses. For example, infection with the Arterivirus porcine reproductive and respiratory syndrome virus (PRRSV) releases the DAMP HMGB1 from infected host cells, resulting in macrophage expression of pro-inflammatory cytokines [15]. Undamaged Influenza A-infected cells release the DAMP S100A9, which activates host inflammatory responses [16]. Influenza A-induced extracellular ATP release may also up-regulate *Streptococcus pneumoniae* virulence genes–providing one possible explanation for the increased lethality of post-influenza pneumococcal pneumonia [17]. Finally, extracellular ATP is both released from Human Immunodeficiency Virus (HIV)-infected macrophages [18] and facilitates HIV entry into new host cells [19]. Therefore, we hypothesized that release of DAMPs from PEDV-infected cells may influence the *C*. *pecorum* developmental cycle. As a first step in testing this hypothesis, we examined the effect of extracellular ATP and adenosine (ADO) on *C*. *pecorum* development.

The aim of this study was to investigate the effect of DAMPs on *C*. *pecorum* inclusion development. Exposure of *Chlamydia*-infected cells to DAMPs reversibly reduced inclusion size, bacteria per inclusion and subsequent chlamydial infectivity upon re-infection of host cells. Notably, aberrant bodies, characteristic of PEDV-induced *C*. *pecorum* persistence, were absent upon DAMP exposure. Parallel evaluation of two chlamydial species, *C*. *pecorum* and *C*. *trachomatis* serovar E, demonstrated that DAMPs exert their effect in a species-specific manner. Comparison of DAMP exposure beginning at 0 hours versus 14 hours post infection showed that similar reduction of inclusions size and lack of induction of aberrant body formation occur independent of the time of initiation of DAMP exposure. This study, to our knowledge, represents the first evaluation of the effects of DAMPs on *C*. *pecorum* inclusion development, and as such provides novel information regarding the host/pathogen interactions of this important, though less frequently studied, chlamydial species.

## Materials and Methods

### Host cells and media

HeLa cells (human cervical adenocarcinoma epithelial cells, CCL-2, American Type Culture Collection, Manassas, VA, USA; kindly provided by Christian Blenn, Institute of Veterinary Pharmacology and Toxicology, University of Zurich, Zurich, Switzerland) were cultured at 37°C and 5% CO_2_ in growth medium for cell propagation and maintenance. Growth medium consisted of Minimal Essential Medium (MEM) with Earle’s salts, 25 mM HEPES, without L-Glutamine (GIBCO, Invitrogen, Carlsbad, CA, USA) supplemented with 10% fetal calf serum (FCS, BioConcept, Allschwil, Switzerland), 4 mM GlutaMAX-I (200 mM, GIBCO), 1% MEM Non-Essential Amino Acids (100x, GIBCO) and 0.2 mg/ml gentamycin (50 mg/ml, GIBCO). For use in experiments, cells were seeded in 24-well plates (Techno Plastic Products AG (TPP), Trasadingen, Switzerland) at a density of 3x10^5^ per well in 1 mL growth medium without gentamycin on 13 mm diameter glass coverslips (Sterilin Limited; Thermo Fisher Scientific, Cambridge, UK) for immunofluorescence (IF) microscopy or transmission electron microscopy (TEM), or directly in the 24-well plates for titration by sub-passage. Infection medium, used for inoculating cells with *Chlamydia*, consisted of growth medium components except FCS and gentamycin. Incubation medium, used to replace infection medium after infection with *Chlamydia*, consisted of growth medium components except gentamycin, supplemented with 1 μg/ml cycloheximide (Sigma-Aldrich, St. Louis, MO, USA) immediately before use. In some experiments, growth medium without cycloheximide was used.

### Reagents

The following reagents were solubilized in sterile distilled H_2_O, stored at -20°C, and thawed immediately before use: adenosine (ADO, Abcam Biochemicals, Cambridge, UK), erythro-9-(2-Hydroxy-3-nonyl)-adenine hydrochloride (EHNA, Sigma-Aldrich), adenosine-5-triphosphate disodium salt (ATP, Sigma-Aldrich), apyrase (Sigma-Aldrich), adenosine-3’,5’-cyclic monophosphate (cAMP, Sigma-Aldrich), 8-Bromo-cAMP (8BrcAMP, Abcam Biochemicals).

### Chlamydial strains

*C*. *pecorum* 1710S (isolate from a swine abortion) was kindly provided by Prof. J. Storz, Baton Rouge, LA, USA [20]. The isolate of the *C*. *trachomatis* strain was originally obtained from S. P. Wang and C. C. Kuo (University of Washington, Seattle, WA, USA) [21]. *C*. *pecorum* and *C*. *trachomatis* were propagated in HeLa cells, and crude stocks were suspended in SPG medium and stored at -80°C. SPG medium consisted of 218 mM sucrose (Sigma-Aldrich), 3.76 mM KH_2_PO4 (Sigma-Aldrich), 7.1 mM K_2_HPO4 (Merck Eurolab AG, Dietlikon, Switzerland) and 5 mM GlutaMAX-100 (GIBCO).

### Study design, infection and exposure of host cells to DAMPs

HeLa cells, cultivated overnight in 24-well plates, were infected with either *C*. *pecorum* or *C*. *trachomatis* at 1 multiplicity of infection (MOI) in 1 ml infection medium and centrifuged for 1 hour (h) at 1000 g and 25°C. After centrifugation, infection medium was replaced by incubation medium and cultures were incubated at 37°C and 5% CO_2_ as previously described [6]. The crude stocks used resulted in approximate infection rates of 40% for *C*. *pecorum* and 20% for *C*. *trachomatis*; this corresponded to approximately 10^7^ recoverable inclusion forming units (IFU) per well for both *C*. *pecorum* and *C*. *trachomatis* diluent-exposed infected cells after 35 or 39 hours of incubation, respectively.

**Fig 1:**
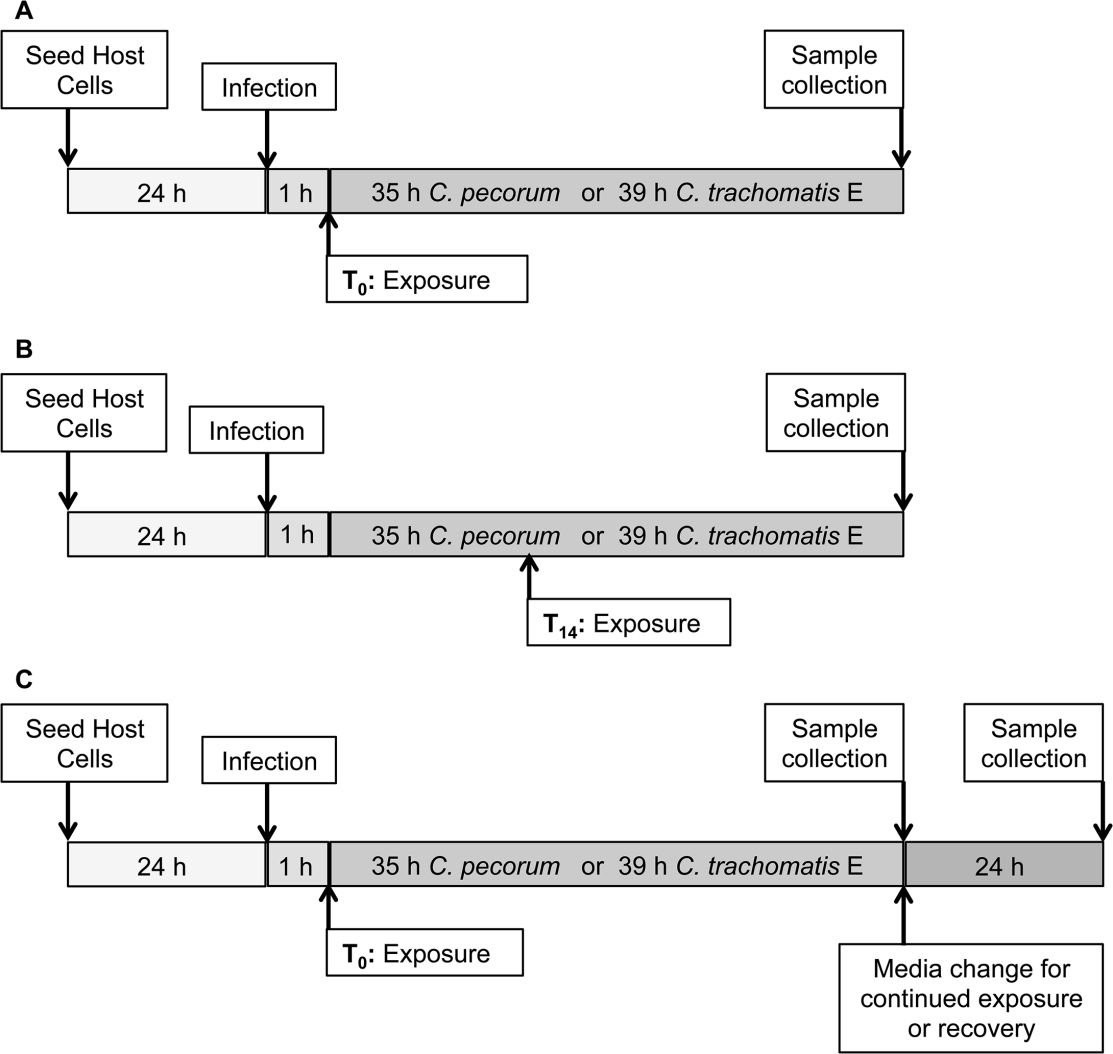
Study Design. (A) Diagram illustrating infection and exposure beginning at T_0_, immediately after infection. Host cells were infected with *Chlamydia* (*C*.) *pecorum* or *C*. *trachomatis* serovar E, exposed to DAMPs in incubation medium, and incubated for 35 or 39 hours, at which time samples were collected for analysis. (B) DAMP exposure beginning at T_14_, 14 hours after infection. DAMPs were added directly to incubation medium. (C) DAMP exposure beginning at T_0_, followed by 35/39-hour incubation, media change for continued DAMP exposure or recovery in the absence of DAMPs, and 24 hours of additional incubation.

**Fig 2:**
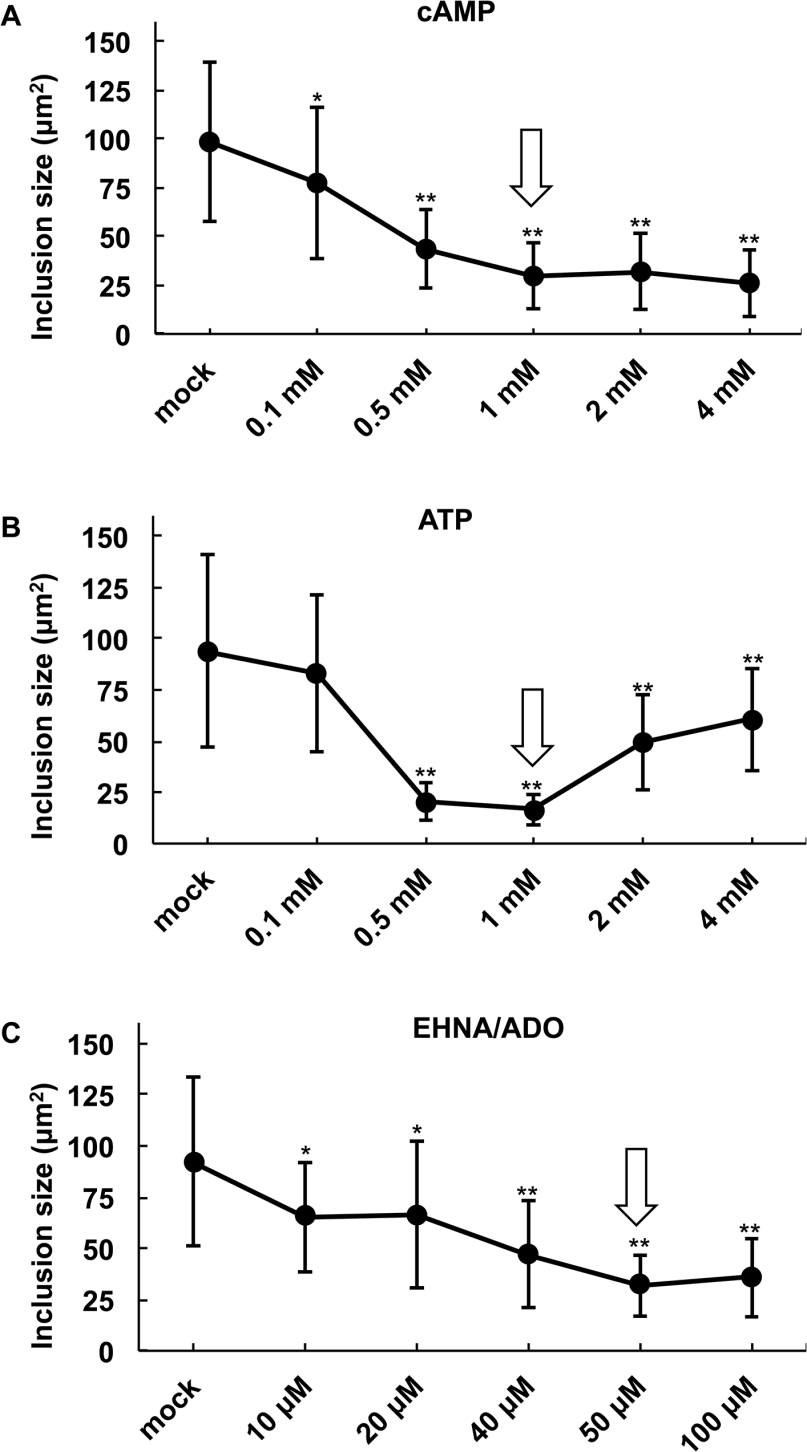
Determination of optimal DAMP concentrations for inhibition of *C*. *pecorum* inclusion development. HeLa cells were infected with *C*. *pecorum* and exposed to the DAMPs (A) cAMP, (B) ATP, or (C) EHNA/ADO (ADO concentration varies, EHNA = 25 μM) in incubation medium immediately after infection. Cells were incubated for 35 hours, fixed and labeled with anti-LPS and DAPI, and inclusion size was determined (mean ± SD; ** p ≤ 0.0001, *p ≤ 0.01, *t* test; n = 50 inclusions per coverslip from a single experiment). Arrows indicate concentrations selected for use in the study.

The effect on *Chlamydia* inclusion formation of various danger associated molecular patterns, DAMPs [cAMP, ATP, ADO], and analogues [8BrcAMP] or modulators [EHNA, apyrase] of these DAMPs, was determined by adding reagents to the incubation medium of infected cells at T_0_, immediately after infection (Fig 1A), or at T_14_, 14 hours after infection (Fig 1B). In a single experiment, the effect of DAMPs added at T_0_ was evaluated in parallel with and without cycloheximide supplementation of the incubation medium. In some experiments, apyrase was added to abrogate the activity of ATP. In these cases, apyrase was added to the incubation medium and the cultures incubated at 37°C and 5% CO_2_ for 15 minutes prior to the addition of ATP. For EHNA/ADO exposure, both reagents were added to the incubation medium together at the indicated times [T_0_ or T_14_]. Diluent alone, sterile distilled H_2_O, served as mock exposure controls. In preliminary experiments for this study, in HeLa cells, *C*. *pecorum* inclusions and host cells begin to burst earlier than *C*. *trachomatis* inclusions and host cells. *Chlamydia-*infected cells were thus incubated until mature inclusions could be evaluated, but bursting of inclusions and cells was not evident: 39 h post infection (hpi) for *C*. *trachomatis*-infected cells, as previously reported [22], and 35 hpi for *C*. *pecorum*-infected cells, as determined by preliminary experiments (Fig 2). In continued exposure and recovery experiments, incubation medium was changed and cells were incubated for a further 24 hours (Fig 1C).

Samples were collected at the indicated times (Fig 1) and processed for further analysis as previously described [6]. For IF microscopy, cells were fixed with absolute methanol (-20°C) for 10 minutes; for TEM cells were fixed with 2.5% glutaraldehyde (Electron Microscopy Sciences, Ft. Washington, USA) for 1 h and embedded in epoxy resin (Fluka; Sigma-Aldrich) by routine methods; for titration by sub-passage infected monolayers were scraped into 1 ml of fresh infection medium and stored at -80°C. For continued exposure or recovery (Fig 1C) sample collection for titration by sub-passage culture supernatant was collected (total volume per well = 1 ml), infected monolayers were scraped into 1 ml of fresh infection medium, and supernatant and scraped cells were pooled before storage at -80°C.

### IF microscopy

*C*. *trachomatis* and *C*. *pecorum* inclusions were visualized using a *Chlamydiaceae* family-specific mouse monoclonal antibody directed against the chlamydial lipopolysaccharide (LPS, Clone ACI-P, 1:200; Progen, Heidelberg, Germany) and 1:500 diluted Alexa Fluor 488-conjugated secondary goat anti-mouse antibody (Molecular Probes, Eugene, OR, USA). Host and chlamydial DNA were labeled using 1 μg/ml 4’, 6-diamidino-2’-phenylindole dihydrochloride (DAPI, Molecular Probes). Coverslips were mounted with FluoreGuard mounting medium (Hard Set; ScyTek Laboratories Inc., Logan, UT, USA) on glass slides and evaluated using a Leica DMLB fluorescence microscope (Leica Microsystems, Wetzlar, Germany) under oil immersion at 1000x magnification with a 1006 objective (PL FLUOTAR 100x/1.30, OIL, ‘/0.17/D, Leica Microsystems) and a 106 ocular objective (Leica L-Plan 10x/ 25 M, Leica Microsystems).

To determine percent of cells infected (inclusions per nucleus) and mean number of nuclei per field (to evaluate possible cell loss from the monolayer), HeLa nuclei and corresponding chlamydial inclusions in each of 8 randomly selected microscopic fields were counted per condition (at least 200 HeLa nuclei per condition). To determine mean inclusion size, 50 randomly selected inclusions were examined per condition and area in μm^2^ was calculated using BonTec measuring and archiving software (BonTec, Bonn, Germany). Representative microscopic images were captured using BonTec software (BonTec) and a UI-2250SE-C-HQ camera (uEye, IDS Imaging Development Systems GmbH, Obersulm, Germany).

### Chlamydial titration by sub-passage

HeLa cells were grown on glass coverslips as described above. Samples were thawed and sonicated on ice for 5 minutes (Branson Sonifier 250; Branson Ultrasonics, Danbury, CT, USA). Sonicated samples were serially diluted in infection medium and infection of the prepared HeLa cells, centrifugation, medium replacement with incubation medium, and incubation was carried out as described for infection of host cells. Fixation and immunostaining was performed as described for IF microscopy. The number of inclusions in 30 random microscopic fields for duplicate coverslips per condition was determined using a Leica fluorescence microscope at 200x magnification with a 206 objective (PL FLUOTAR 20x/0.50 PH 2, ‘/0.17/B) and a 106 ocular objective (Leica L-Plan 10x/25 M, Leica Microsystems). Inclusion forming units (IFU) per ml of undiluted inoculum was then calculated according to previously published methods [4].

### TEM

Ultrathin (80 nm) sections were mounted on gold grids (Merck), contrasted with uranyl acetate dehydrate (Fluka; Sigma-Aldrich) and lead citrate (Merck). The sections were evaluated using a Philips CM10 electron microscope (Software release version 5.1; FEI Company, Hillsboro, OR, USA) and imaged using a Gatan Orius SC 1000 CCD Camera with software version Digital Micrograph 2.30 (Gatan Inc., Warrendale, PA, USA). Images were analyzed using Photoshop CS6 software (Adobe Systems Incorporated, San Jose, CA, USA). To determine mean number of bacteria per inclusion, the total number of bacteria in ten inclusions per condition was counted. Bacterial morphology was determined as previously described [22]: EB (dark, 0.25–0.5 μm), IB (dark center and pale periphery), RB (pale, 0.5–1 μm) and AB (pale, <2 μm).

### Statistical analysis

Statistical analyses were performed using Microsoft Excel. Significance of the difference of means was determined by unpaired *t* test and p values of <0.05 were considered significant. P values were confirmed using the GraphPad QuickCalcs Web site: http://www.graphpad.com/quickcalcs/ttest1/ (accessed June 2014). Unless stated otherwise, results are displayed as means, +/- standard deviation, of the results from three independent experiments.

## Results

### cAMP, ATP and EHNA/ADO inhibit *Chlamydia* inclusion development

To characterize the effect of the damage/danger associated molecular patterns (DAMPs) extracellular ATP and adenosine (ADO) on *C*. *pecorum* inclusion development, we performed a preliminary experiment to determine optimal DAMP doses for inhibition of chlamydial inclusion formation in HeLa cells. Cyclic AMP (cAMP) has not been traditionally categorized as a DAMP, but rather an intracellular “second messenger” downstream of cell surface receptor stimulation. However, several studies suggest that cAMP can egress from and bind receptors on various cell types, exerting subsequent influence on cellular function (reviewed in [23]). Furthermore, cAMP levels quickly increase in HeLa cells after ADO stimulation, and both cell-permeant and non-cell-permeant cAMP have been shown to inhibit chlamydial development [14,24,25]. Therefore, we also evaluated the effect of cAMP on *C*. *pecorum* inclusion development, and refer to it here as a DAMP.

Cells were infected with *C*. *pecorum* at MOI 1 and the infected cells were then immediately exposed to various concentrations of cAMP or ATP (range from 0.1 to 4 mM) or ADO (range from 10 to 100 μM, with 25 μM EHNA) added directly to the incubation medium as a single dose (Fig 1A). The incubation medium was allowed to remain on the infected cells until 35 hours post infection (hpi), when the cells were fixed and immunolabelled with anti-chlamydial LPS antibodies followed by mean inclusion size determination by fluorescence microscopy (Fig 2). Inclusion size was evaluated as a measure of inhibition of inclusion development because, in general, relatively small inclusions are considered characteristic of the chlamydial persistence/stress response (reviewed in [26]).

Exposure to cAMP reduced inclusion size in a dose dependent manner (Fig 2A). cAMP was used at 1 mM in our subsequent experiments because this was the lowest concentration resulting in maximum inclusion size reduction to approximately 30% of control size. Exposure to ATP caused significant reduction in inclusion size in the range of 0.5 to 4 mM concentration; however, a typical dose dependent response was not observed (Fig 2B). Instead, relatively low concentrations of ATP decreased inclusion size, while higher concentrations of ATP failed to decrease inclusion size. Maximal reduction to less than 20% of control size was achieved with 1 mM ATP, and this concentration was used in subsequent experiments.

EHNA, at 25 μM, was previously shown to potentiate the anti-chlamydial activity of various concentrations of ADO [14,27]; therefore, we evaluated the effect on inclusion size of a range of ADO concentrations in the presence of 25 μM EHNA. Exposure to ADO to *C*. *pecorum*-infected cells reduced inclusion size in a dose dependent manner (Fig 2C). ADO at 50 μM, in the presence of 25 μM EHNA (EHNA/ADO), resulted in a maximal reduction to approximately 35% of control inclusion size, and was used in subsequent experiments.

Previous studies evaluating exposure of *Chlamydia*-infected HeLa cells to 1 mM cAMP, ATP or ADO for 48 hours or 50 μM ADO plus 25 μM EHNA for 42 hours did not report evidence of abnormal host cell appearance or cytotoxicity [14,24]. Microscopic evidence of apoptosis caused by DAMPs, for example exposure of cultured immune cells to 1mM ATP, includes characteristic nuclear condensation and/or fragmentation, which can be observed by IF microscopy [28,29]. We observed no such IF microscopic evidence of abnormal nuclear appearance upon 35 hours of exposure of *C*. *pecorum*-infected HeLa cells to any evaluated concentrations of cAMP, ATP, or EHNA/ADO. This observation is not surprising since chlamydial infection has been demonstrated to inhibit both extrinsic and intrinsic host cell apoptosis pathways [30].

### cAMP, ATP and EHNA/ADO reduce mean *Chlamydia* inclusion size and number of bacteria per inclusion, but do not induce aberrant body formation

**Fig 3:**
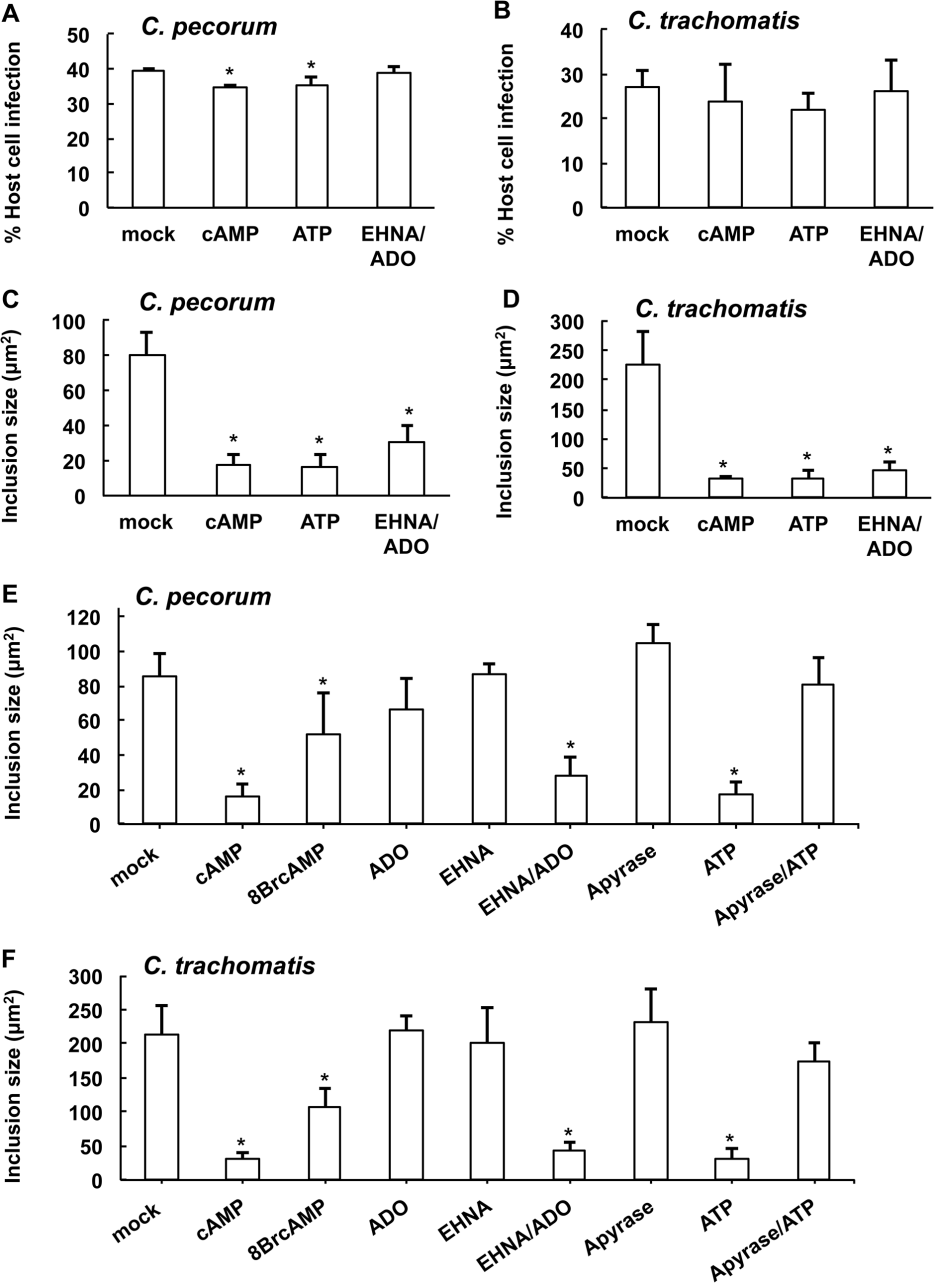
DAMPs reduce chlamydial inclusion size. HeLa cells were infected with *C*. *pecorum* or *C*. *trachomatis* serovar E and exposed to cAMP (1 mM), 8BrcAMP (1 mM), ATP (1 mM), apyrase (2.5 U), apyrase (2.5 U) followed by ATP (1 mM), ADO (50 μM), EHNA (25 μM), or ADO (50 μM) plus EHNA (25 μM) in incubation medium immediately after infection. Cells were incubated for 35 hours (*C*. *pecorum*) or 39 hours (*C*. *trachomatis*), fixed and labeled with anti-LPS and DAPI. Number of inclusions per nucleus was determined and percent infection was calculated for *C*. *pecorum* (A) and *C*. *trachomatis* (B) (mean ± SD; *p ≤ 0.05, *t* test; n = 3). Mean inclusion size was determined for *C*. *pecorum* (C and E) and *C*. *trachomatis* (D and F) (mean ± SD; *p ≤ 0.05, *t* test; n = 3).

In subsequent experiments (Fig 3), exposure of *Chlamydia*-infected HeLa cells to DAMPs allowed robust infection of host cells. The addition of cAMP or ATP caused only a small, albeit statistically significant, decrease in percent of host cells infected by *C*. *pecorum* (Fig 3A) compared to mock treatment. The percent of *C*. *pecorum*-infected cells was reduced to 34.4% by cAMP (p = 0.0016) and to 35.2% by ATP (p = 0.0362), compared to the control infection of 39.6%. Reduction in percent of cells infected was not observed for EHNA/ADO treated *C*. *pecorum*-infected cells (Fig 3A) or for any treated groups of *C*. *trachomatis*-infected cells (Fig 3B). The decrease in percent infection of host cells by *C*. *pecorum* in the presence of cAMP or ATP, though statistically significant, likely represents the small size of these inclusions reducing reticle-delimited, field-based microscopic detection. Thus we expect this small reduction in infectivity is unlikely to be biologically relevant.

Exposure to 1 mM cAMP or ATP, or 50 μM ADO in the presence of 25 μM EHNA, of *C*. *pecorum-* or *C*. *trachomatis*-infected HeLa cells, reduced mean inclusion size (Fig 3), as expected based on results seen in the preliminary dose experiment (Fig 2). The average *C*. *pecorum* inclusion size was reduced to 22.0% of control with the addition of cAMP, 20.3% of control with the addition of ATP, and 38.9% of control with the addition of EHNA/ADO (Fig 3C). Similarly, and to a larger degree, the average *C*. *trachomatis* inclusion size was reduced to 13.8% of control with the addition of cAMP, 14.0% of control with the addition of ATP, and 20.0% of control with the addition of EHNA/ADO (Fig 3D).

**Fig 4:**
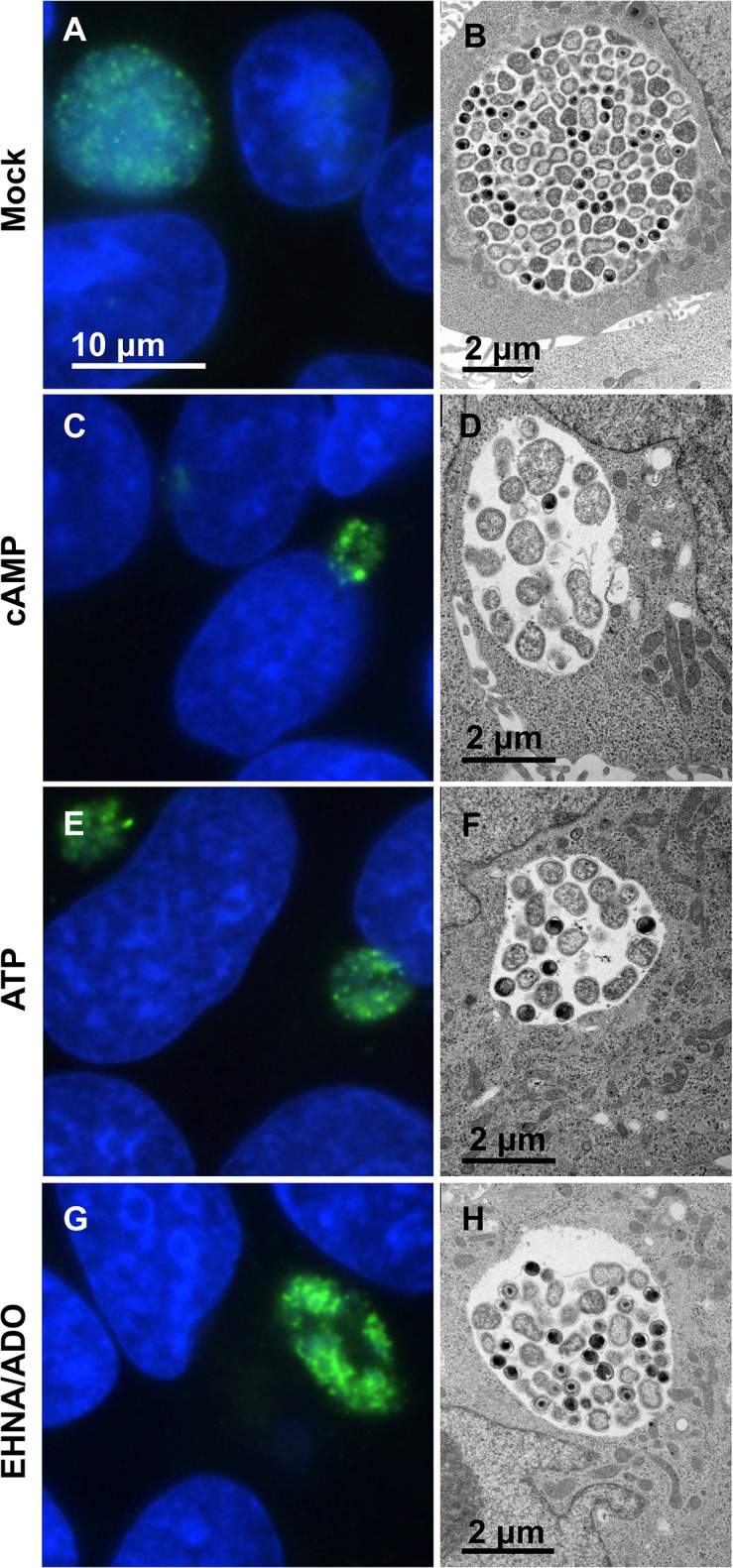
cAMP, ATP, and EHNA/ADO inhibit *C*. *pecorum* inclusion development without inducing aberrant body formation. HeLa cells were infected with *C*. *pecorum* and exposed to the DAMPs cAMP (1 mM), ATP (1 mM), or ADO (50 μM, plus 25 μM EHNA) in incubation medium immediately after infection. Cells were incubated for 35 hours and fixed for further processing. Cells were labeled with anti-LPS (green) and DAPI (blue) for immunofluorescence microscopic analysis (left). Cells were processed by standard methods for transmission electron microscopic analysis (right). Representative images are shown for *C*. *pecorum*-infected mock- (A and B), cAMP- (C and D), ATP- (E and F), and EHNA/ADO-exposed (G and H) cells. Immunofluorescence microscopy images represent one experiment of three independently repeated experiments with similar results. Transmission electron microscopy images represent a single experiment.

**Fig 5:**
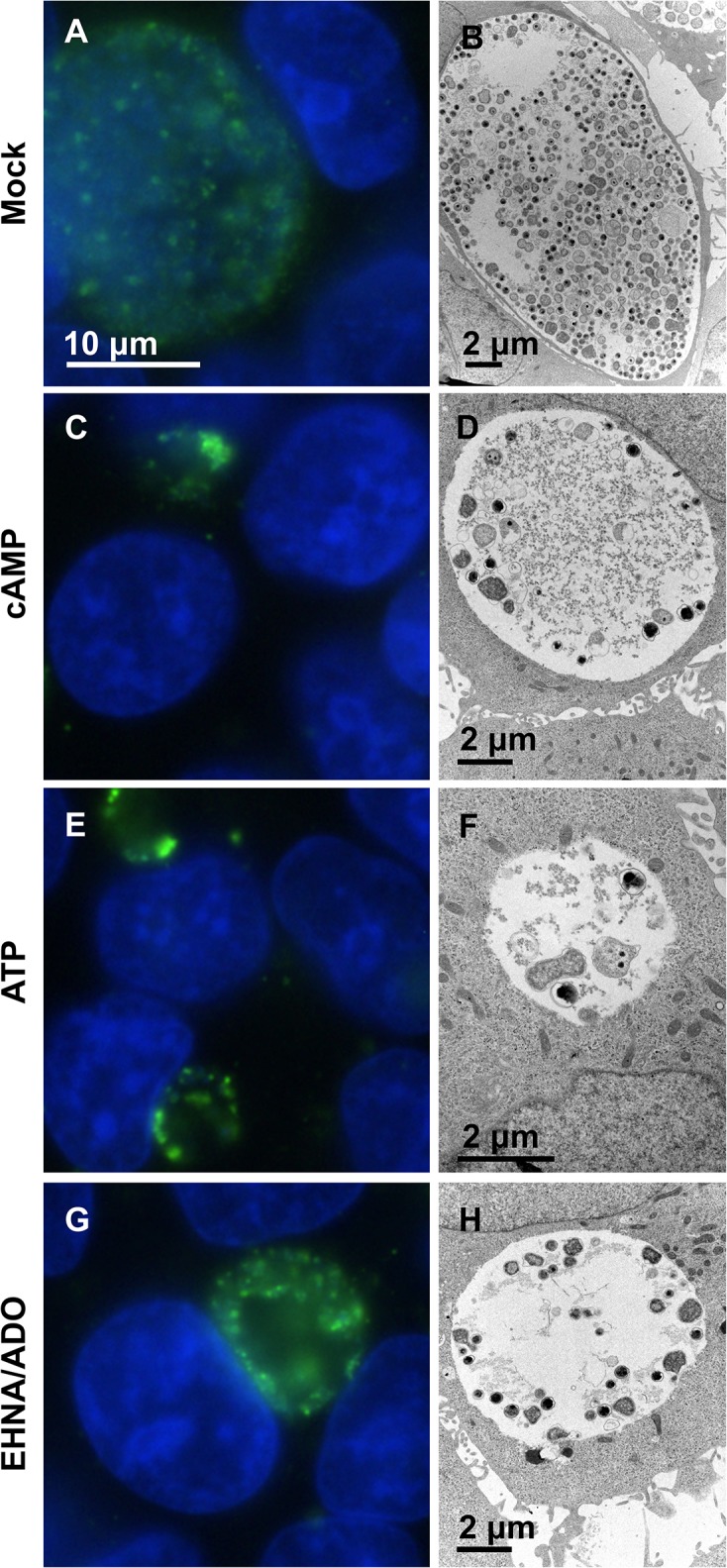
cAMP, ATP, and EHNA/ADO inhibit *C*. *trachomatis* inclusion development without inducing aberrant body formation. HeLa cells were infected with *C*. *trachomatis* serovar E and exposed to the DAMPs cAMP (1 mM), ATP (1 mM), or ADO (50 μM, plus 25 μM EHNA) in incubation medium immediately after infection. Cells were incubated for 39 hours and fixed for further processing. Cells were labeled with anti-LPS (green) and DAPI (blue) for immunofluorescence microscopic analysis (left). Cells were processed by standard methods for transmission electron microscopic analysis (right). Representative images are shown for *C*. *trachomatis*-infected mock- (A and B), cAMP- (C and D), ATP- (E and F), and EHNA/ADO-exposed (G and H) cells. Immunofluorescence microscopy images represent one experiment of three independently repeated experiments with similar results. Transmission electron microscopy images represent a single experiment.

**Fig 6:**
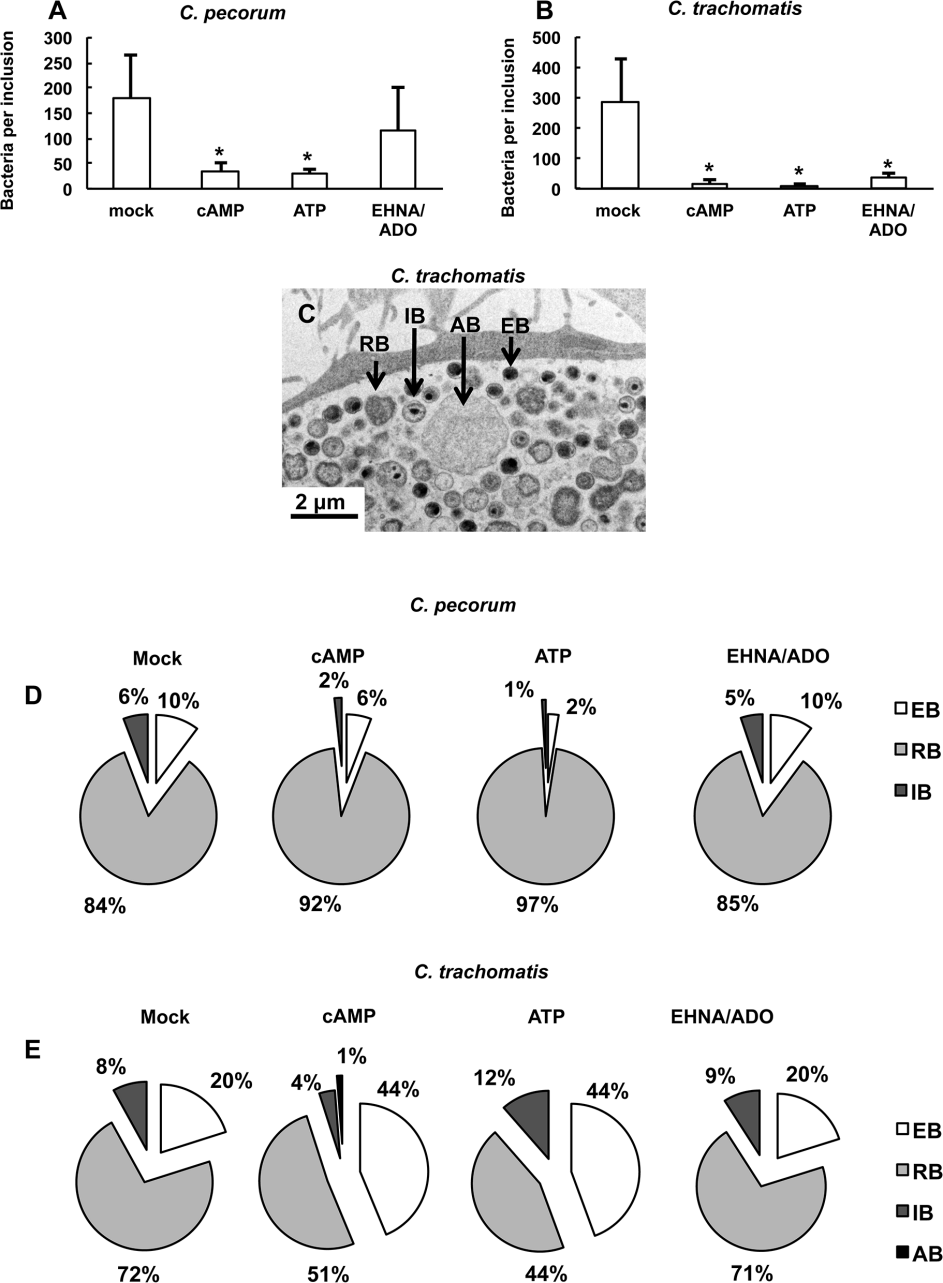
cAMP, ATP and EHNA/ADO reduce the number of bacteria per inclusion. HeLa cells were infected with *C*. *pecorum* or *C*. *trachomatis* serovar E and exposed to the DAMPs cAMP (1 mM), ATP (1 mM), or ADO (50 μM, plus 25 μM EHNA) in incubation medium immediately after infection. Cells were incubated for 35 hours (*C*. *pecorum*) or 39 hours (*C*. *trachomatis*), fixed, and processed by standard methods for TEM analysis. Total number of bacteria (EB, IB, RB, and AB) in ten inclusions per condition was determined, and mean number of bacteria per inclusion (A and B) was calculated (mean ± SD; *p ≤ 0.0001, *t* test; n = 10 inclusions from a single experiment). A representative image of an inclusion in mock-exposed, *C*. *trachomatis*-infected cells (C) shows EB (dark, 0.25–0.5 μm), IB (dark center and pale periphery), RB (pale, 0.5–1 μm) and AB (pale, <2 μm). Analyses of the mean relative proportion of EB, RB, IB and AB per each experimental group are shown for *C*. *pecorum* (D) and *C*. *trachomatis* (E).

Immunofluorescence (IF) and transmission electron microscopy (TEM) showed that *C*. *pecorum* (Fig 4) and especially *C*. *trachomatis* (Fig 5) inclusions exposed to DAMPs were not only smaller, but contained sparsely distributed chlamydial bodies within the inclusions, compared to *C*. *pecorum* and *C*. *trachomatis* controls. Additionally, quantitative analysis of TEM images indicated that exposure to DAMPs reduced the mean number of bacteria per inclusion in *C*. *pecorum* and *C*. *trachomatis*-infected HeLa cells (Fig 6A and 6B). The mean bacteria per *C*. *pecorum* inclusion was reduced to 19.1% of control with the addition of cAMP, 16.9% of control with the addition of ATP, and 64% of control with the addition of EHNA/ADO, although the reduction caused by EHNA/ADO failed to reach statistical significance (Fig 6A). The mean bacteria per *C*. *trachomatis* inclusion was reduced, similarly and to a larger extent, to 5.8% of control with the addition of cAMP, 3.8% of control with the addition of ATP, and 12.2% of control with the addition of EHNA/ADO (Fig 6B).

Cycloheximide was included in the incubation medium for experiments presented in Figs 2–6, limiting host cell *de novo* protein synthesis and facilitating chlamydial inclusion growth and infectious EB production [31]. To evaluate the impact of host cell *de novo* protein synthesis on DAMP-dependent modulation of inclusion development, DAMPs were added at T_0_, either with or without cycloheximide supplementation of the incubation medium (S1 Fig). IF microscopy showed, as expected, that chlamydiae (S1A and S1Q Fig, *C*. *pecorum*, 90 μm^2^; S1I and S1S Fig, *C*. *trachomatis*; 375 μm^2^) cultured in the presence of cycloheximide develop larger inclusions than do chlamydiae cultured in the absence of cycloheximide (S1E and S1R Fig, *C*. *pecorum*, 37 μm^2^; S1M and S1T Fig, *C*. *trachomatis*, 131 μm^2^). Each DAMP significantly reduced inclusion size compared to the control, in both chlamydial species evaluated, both in the presence and absence of cycloheximide. However, the DAMP-dependent effect was relatively robust in the presence of cycloheximide compared to in the absence of cycloheximide. Mean *C*. *pecorum* inclusion size was reduced, by all DAMPs evaluated, to less than 25% of control inclusion size in the presence of cycloheximide (S1A–S1D and S1Q Fig), but to less than 65% of control inclusion size in the absence of cycloheximide (S1E–S1H and S1R Fig). Similarly, mean *C*. *trachomatis* inclusion size was reduced, by all DAMPs evaluated, to less than15% of control inclusion size in the presence of cycloheximide (S1I–S1L and S1S Fig), but to less than 40% of control inclusion size in the absence of cycloheximide (S1M–S1P and S1T Fig).

An increase in the presence of aberrant bodies (AB), which can be recognized by IF microscopy and TEM as abnormally large chlamydial forms within inclusions [6] (Fig 6C), was not observed by IF microscopy upon DAMP exposure of cells infected with either *C*. *pecorum* (Fig 6D, S2A Fig) or *C*. *trachomatis* (Fig 6E, S2B Fig). TEM analyses of the mean relative proportions of EB, RB, IB (intermediate bodies, differentiating bacterial form intermediate to RB and EB) and AB indicate that cAMP and ATP exposure, but not EHNA/ADO exposure, significantly altered the relative proportion of bacterial developmental forms (Fig 6D and 6E, S2 Fig). Exposure of *C*. *pecorum*-infected cells (Fig 6D, S2A Fig) to cAMP and ATP reduced the proportion of EB to 6% and 2%, respectively, compared to the control proportion of 10% (although this reduction was not statistically significant for cAMP exposure). A similar effect was seen for *C*. *pecorum* IB, which showed a reduced proportion of 2% and 1%, upon cAMP and ATP exposure, respectively, compared the control proportion of 6%. This was accompanied by a cAMP- and ATP-dependent increase in the proportion of *C*. *pecorum* RB to 92% and 97% respectively, compared to the control proportion of 84%. In marked contrast, exposure of *C*. *trachomatis*-infected cells (Fig 6E, S2B Fig) to cAMP and ATP increased the proportion of EB to 44% (for both DAMPs), compared to the control proportion of 20%; this was accompanied by a cAMP- and ATP-dependent decrease in the proportion of RB to 51% and 44% respectively, compared to the control proportion of 72% (no effect on IB proportion was observed). EHNA/ADO had no effect on the proportion of chlamydial developmental forms for either species of *Chlamydia* evaluated (Fig 6D and 6E, S2A and S2B Fig). Electron-dense granular material (Fig 5F) was observed by TEM in multiple inclusions of all treatment groups, including untreated controls, of *C*. *trachomatis*-infected cells. This material appeared to be more prevalent in DAMP-exposed *C*. *trachomatis*-infected groups than in the corresponding control group. A single incident of a similar material in an inclusion of *C*. *pecorum*-infected cells was observed (cAMP-treated group).

### The effect of ATP and ADO on *Chlamydia* inclusion size can be modulated by apyrase and EHNA, respectively; the effect of cAMP can be reproduced by cell-permeant 8BrcAMP

Modulators and analogues of the DAMPs evaluated were used to confirm that the effects observed were attributable to these DAMPs. EHNA is an ADO deaminase inhibitor [32]. ADO deaminase irreversibly deaminates ADO to inosine, so EHNA maintains relatively high ADO levels by preventing its deamination, and thus helps maintain extracellular adenosine signaling. To confirm that the inhibition of inclusion formation observed with exposure to ADO was due to the activity of the exogenous ADO, and not activity of EHNA alone or other off-target effects, we evaluated the effect of ADO and EHNA, separately and in combination, on *Chlamydia* inclusion size (Fig 3E and 3F). As expected, we found that EHNA potentiated the reduction of inclusion size by ADO in both *Chlamydia* species evaluated. Exposure to EHNA or ADO alone had no significant effect on *C*. *pecorum* or *C*. *trachomatis* inclusion size.

Apyrase is an enzyme that catalyzes the hydrolysis of ATP to AMP. Thus apyrase should reduce effects caused by action of exogenous ATP. Apyrase has previously been shown to abrogate the inhibitory effect of extracellular ATP on *C*. *trachomatis* L2 inclusion formation [27]. To confirm that the inhibitory effect on inclusion formation observed upon exposure to ATP was due to the activity of the extracellular ATP, we evaluated the effect of apyrase on ATP-dependent effects (Fig 3E and 3F). As expected, apyrase abrogated the ATP-dependent reduction of inclusion size in both *Chlamydia* species evaluated. In the presence of apyrase, exposure to exogenous ATP reduced inclusion size compared to mock treated controls, but this reduction did not reach statistical significance, in contrast to exposure to ATP alone. Furthermore, exposure of *Chlamydia*-infected cells to apyrase alone increased inclusion size compared to mock treated controls, however this increase did not reach statistical significance.

Previously, cAMP levels were found to be increased in *C*. *trachomatis* L2-infected HeLa cells, and exposure to a cell-permeant analogue of cAMP, 8-Bromo-cAMP (8BrcAMP), was subsequently shown to have an inhibitory effect on *C*. *trachomatis* L2 inclusion formation in HeLa cells, as measured by a reduction in relative *C*. *trachomatis* L2 16S rRNA levels [14]. This was in contrast to a previous report that indicated exposure to cAMP, but not 8BrcAMP, reduced inclusion size in *C*. *trachomatis* L2-infected HeLa cells [24]. We compared the effect of cAMP and 8BrcAMP on inclusion development, as measured by mean inclusion size (Fig 3E and 3F), and found that exposure of *C*. *pecorum*- and *C*. *trachomatis* E-infected HeLa cells to 8BrcAMP significantly reduced inclusion size compared to mock exposed controls. This was similar to, though less dramatic than, the observed effect of non-cell-permeant cAMP.

To further confirm that DAMP exposure did not exert the effects observed by causing significant host cell death, we evaluated DAMP-exposed *Chlamydia*-infected HeLa cells for cell loss from the monolayer, and mean nuclei per field was determined (S1A–S1D Fig). Exposure of *C*. *pecorum-* or *C*. *trachomatis*-infected HeLa cells to DAMPs did not result in a reduction of host cell nuclei numbers compared to control values. There was no evidence of cytotoxicity by IF microscopy or TEM. Similarly, no cell loss from the monolayer was observed (S1E–S1G Fig) at any DAMP concentration evaluated in the preliminary dose experiment (Fig 2).

### cAMP, ATP, and EHNA/ADO reversibly reduce chlamydial inclusion size and infectivity in a species-specific manner

**Fig 7:**
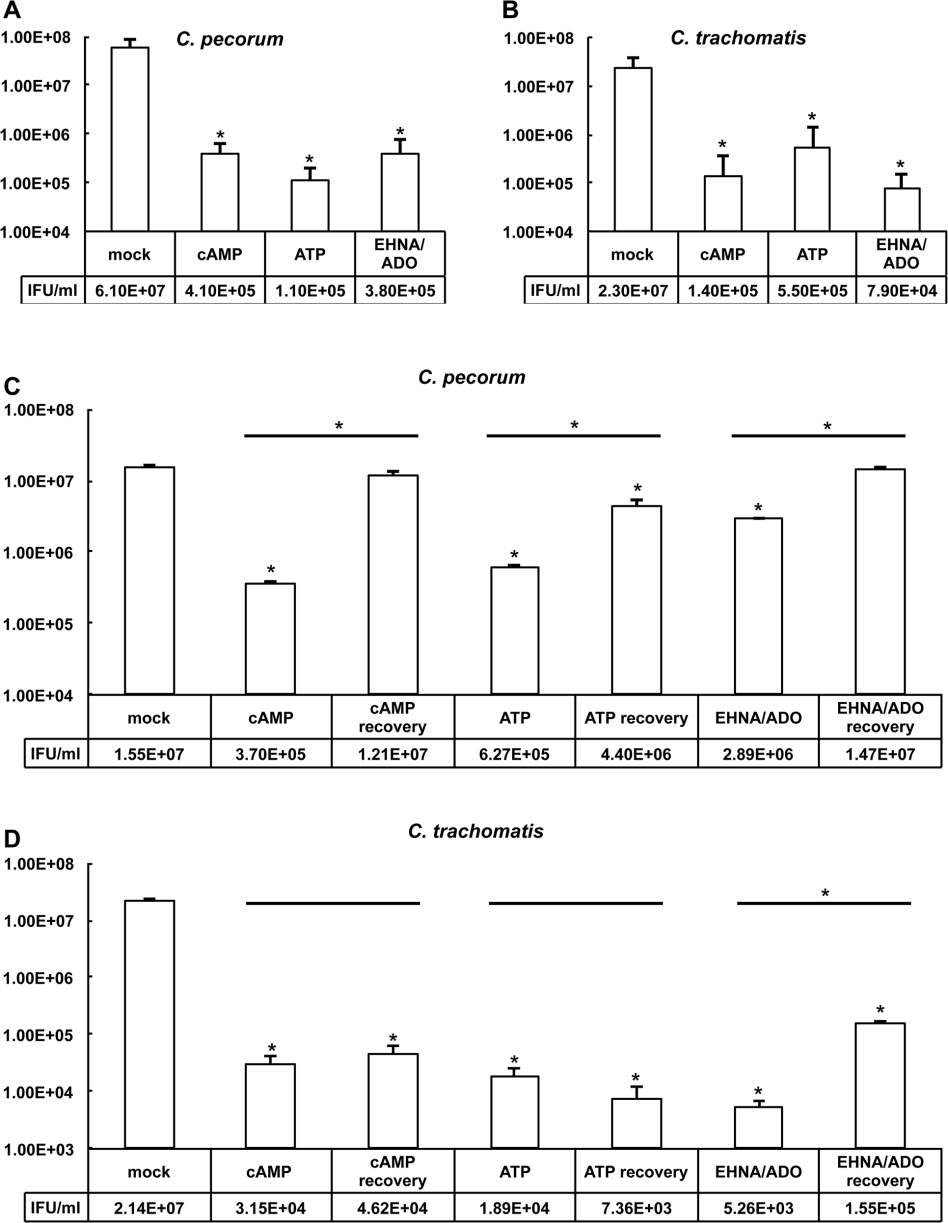
DAMPs reversibly reduce chlamydial infectivity in a species-specific manner. HeLa cells were infected with *C*. *pecorum* or *C*. *trachomatis* serovar E and exposed to cAMP (1 mM), ATP (1 mM), or ADO (50 μM, plus 25 μM EHNA) in incubation medium immediately after infection and incubated for 35 hours (*C*. *pecorum*) or 39 hours (*C*. *trachomatis*). Infected monolayers were then collected and processed for titration by sub-passage. Number of inclusions was determined and inclusion forming units (IFU) per ml (IFU/ml) was calculated for *C*. *pecorum* (A) and *C*. *trachomatis* (B) (mean ± SD; *p ≤ 0.05, *t* test; n = 3). In some wells, incubation medium on cells was replaced with fresh incubation medium with DAMPs for continued exposure or without DAMPs for recovery. These cells were incubated a further 24 hours before processing for titration by sub-passage. Number of inclusions was determined and IFU/ml was calculated for *C*. *pecorum* (C) and *C*. *trachomatis* (D) (mean ± SD, *p ≤ 0.05, *t* test; values are derived from duplicate determinations within a representative experiment, and represent results obtained from two independent experiments).

To evaluate the impact of DAMP exposure of *Chlamydia*-infected cells on subsequent infectious EB production, infected and exposed cells were collected and subjected to freeze/thaw and sonication to lyse cells and release EB. These samples were then used to re-infect HeLa cells, and inclusion forming units (IFU) per ml were determined (Fig 7A and 7B). As expected, based on the marked reduction in inclusion size upon DAMP exposure of *Chlamydia*-infected cells (Fig 3C and 3D), and the reduction of bacteria per inclusion associated with this reduction in inclusion size (Fig 6A and 6B), IFU/ml was significantly reduced in all DAMP exposed groups, compared to the control exposed group, for both *C*. *pecorum* (Fig 7A) and *C*. *trachomatis* E (Fig 7B). cAMP, ATP, and EHNA/ADO exposure reduced IFU/ml to less than 1% of the control in all DAMP-exposed *C*. *pecorum*-infected cells (Fig 7A), to less than 1% of the control in cAMP and EHNA/ADO-exposed *C*. *trachomatis*-infected cells, and to less than 3% of the control in ATP-exposed *C*. *trachomatis*-infected cells (Fig 7B). Notably, though DAMP exposure decreased chlamydial infectious EB production by at least 97% (Fig 7), reduction in inclusion size ranged from only approximately 61% to 86% of control values for both species tested (Fig 3), suggesting that there is not a strong correlation between inclusion size and infectious EB production.

Cycloheximide was included in the incubation medium for the experiment presented in Fig 7A and 7B, and consequently the marked DAMP-dependent reduction of infectious EB production for both chlamydial species tested occurred specifically under the condition of limited host cell *de novo* protein synthesis. To investigate the impact of this restriction on the observed DAMP-dependent anti-chlamydial effect, DAMPs were added at T_0_, either with or without cycloheximide supplementation of the incubation medium, and processed to determine infectious EB production, presented as IFU/ml (S4 Fig). In contrast to the similar, but less substantial, DAMP-dependent reductive effect on inclusions size noted in the absence of cycloheximide versus the presence of cycloheximide (S1 Fig), reduction, and even reversal, of the DAMP-dependent reductive effect on infectious EB production was observed in the absence of cycloheximide versus the presence of cycloheximide (S4 Fig). *C*. *pecorum* IFU/ml, as expected, was reduced to less than 1% of the control IFU/ml by all DAMPs evaluated in the presence of cycloheximide (S4A Fig). However, in the absence of cycloheximide, EHNA/ADO exposure reduced *C*. *pecorum* IFU/ml to only 73.5% of the control IFU/ml, while cAMP and ATP exposure increased *C*. *pecorum* IFU/ml to 311.2% and 271.8%, respectively (S4B Fig). *C*. *trachomatis* IFU/ml, was reduced to less than 1% of the control IFU/ml, in the presence of cycloheximide, upon exposure to all DAMPs evaluated (S4C Fig). In the absence of cycloheximide, however, *C*. *trachomatis* IFU/ml was reduced to 12.4% of control by EHNA/ADO, not significantly changed by cAMP (though a reduction to 74.6% of control IFU/ml closely approached, but did not reach, significance; p = 0.0540) and increased to 163.3% of control by ATP (S4D Fig).

Previous reports indicate that cAMP, ATP, or EHNA/ADO cause reversible inhibition of *C*. *trachomatis* (serotypes L2 and/or D) inclusion development in HeLa cells [14,24,27]; while ATP-induced inhibition of *Chlamydia (C*.*) muridarum* or *C*. *psittaci* inclusion development in macrophages has not been described as reversible [11–13]. To determine if the anti-chlamydial effects of DAMPs observed here in *C*. *pecorum*- and *C*. *trachomatis*-infected HeLa cells are reversible, we repeated the 35 and 39 hour exposure of *C*. *pecorum*- and *C*. *trachomatis* E infected-HeLa cells to DAMPs, then changed the incubation medium, replacing it with either incubation medium alone (recovery condition) or incubation medium containing cAMP, ATP, or EHNA/ADO (continued exposure condition; using same concentrations as initial 35–39 hour exposures) and incubated the infected cells a further 24 hours (Fig 1C).

**Fig 8:**
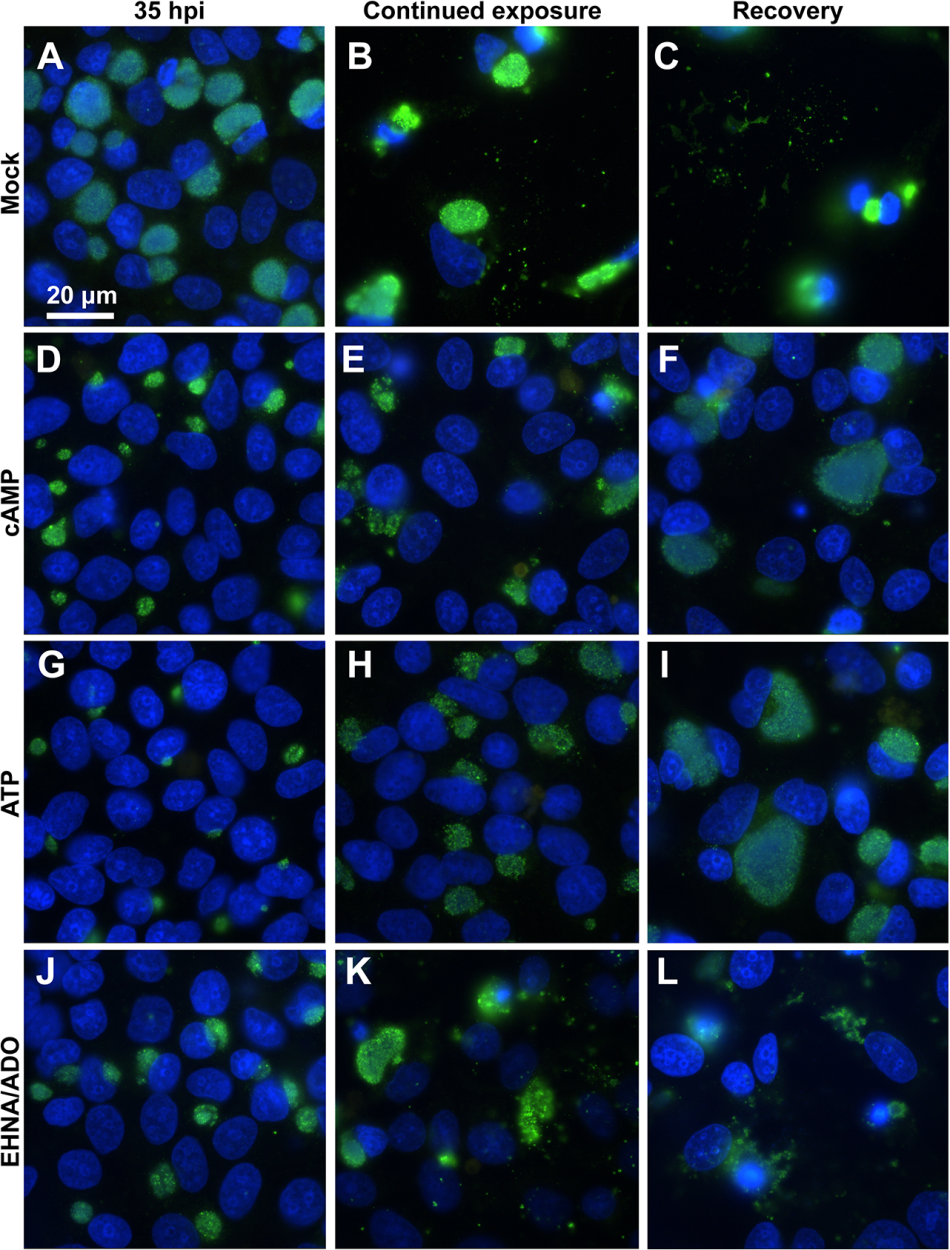
cAMP, ATP and EHNA/ADO reversibly reduce *C*. *pecorum* inclusion size. HeLa cells were infected with *C*. *pecorum* (A-L) and exposed to cAMP (1 mM; D-F), ATP (1 mM; G-I), or ADO (50 μM, plus 25 μM EHNA; J-L) in incubation medium immediately after infection and incubated for 35 hours (A-C = mock exposed). At 35 hours post infection cells were fixed and labeled with anti-LPS (green) and DAPI (blue) (left). In replicate wells, incubation medium on cells was replaced with fresh incubation medium with DAMPs for continued exposure (middle) or without DAMPs for recovery (right). These cells were incubated a further 24 hours before fixation and labeling with anti-LPS and DAPI. Images represent one experiment of two independently repeated experiments with similar results.

IF microscopic analysis of *C*. *pecorum*-infected cells (Fig 8) demonstrated that, for all DAMP-exposed groups (Fig 8D–8L), excluding the mock-exposed control (Fig 8A–8C), inclusions increased in size from 35 hpi (Fig 8 left) to 24 hours later (59 hpi), despite continued exposure to DAMPs (Fig 8 middle). Mock-exposed cells, as expected, were almost entirely lost from the coverslip monolayer by 59 hpi (Fig 8B and 8C). The apparent density of bacteria within the *C*. *pecorum* inclusions, reduced by the initial 35-hour exposure to cAMP, ATP or EHNA/ADO, however, did not substantially increase visibly upon 24 hours of continued exposure.

In contrast, in *C*. *pecorum*-infected cells exposed to cAMP or ATP for 35 hours then allowed to recover in the absence of DAMPs for 24 hours (Fig 8F and 8I), both the size and visible bacterial density of inclusions increased substantially compared to similar infected cells subjected to continued DAMP exposure (Fig 8E and 8H). However, *C*. *pecorum*-infected cells exposed to EHNA/ADO for 35 hours then allowed to recover in the absence of DAMPs for 24 hours (Fig 8L) showed marked cell loss, apparently ruptured inclusions and abundant extracellular chlamydial bodies, making comparison of inclusion size upon continued DAMP exposure versus recovery unreliable in EHNA/ADO-exposed *C*. *pecorum*-infected cells. Furthermore, *C*. *pecorum*-infected, EHNA/ADO-exposed cells were also lost from the monolayer after 24 hours of continued exposure following the initial 35-hour exposure, and extracellular chlamydial bodies were apparent (Fig 8K).

**Fig 9:**
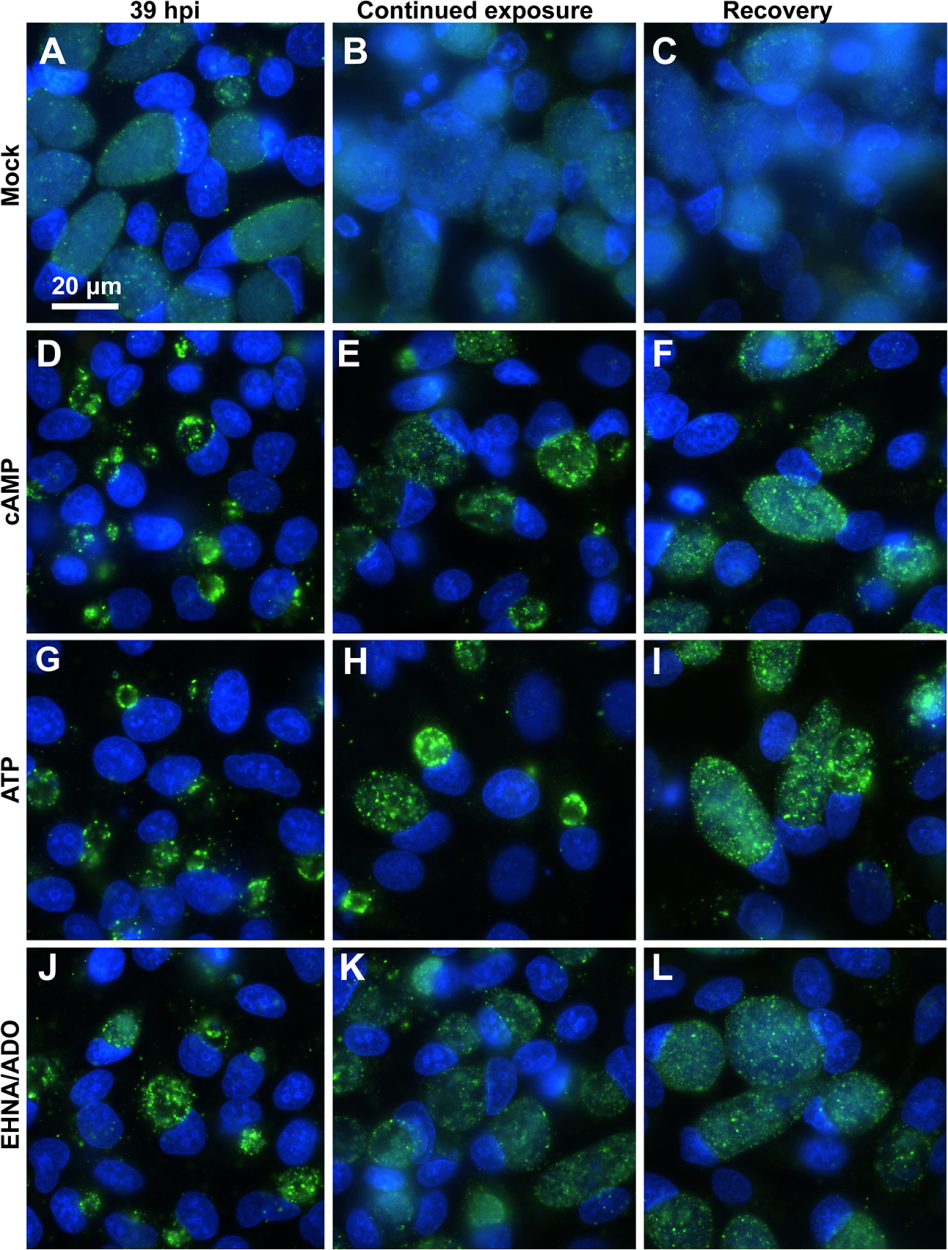
cAMP, ATP and EHNA/ADO reversibly reduce *C*. *trachomatis* inclusion size. HeLa cells were infected with *C*. *trachomatis* serovar E (A-L) and exposed to cAMP (1 mM; D-F), ATP (1 mM; G-I), or ADO (50 μM, plus 25 μM EHNA; J-L) in incubation medium immediately after infection and incubated for 39 hours (A-C = mock exposed). At 39 hours post infection cells were fixed and labeled with anti-LPS (green) and DAPI (blue) (left). In replicate wells, incubation medium on cells was replaced with fresh incubation medium with DAMPs for continued exposure (middle) or without DAMPs for recovery (right). These cells were incubated a further 24 hours before fixation and labeling with anti-LPS and DAPI. Images represent one experiment of two independently repeated experiments with similar results.

In mock-exposed *C*. *trachomatis*-infected cells, less marked cell loss from the monolayer was observed upon 24 hours of additional culture after 39 hpi than was observed for corresponding mock-exposed *C*. *pecorum*-infected cells (Fig 9B and 9C). However, burst cells and cell fragments adherent to the host cell monolayer surface were visible on all areas of the coverslip (fluorescent signal from the nuclei can be seen, Fig 9B and 9C). In *C*. *trachomatis*-infected HeLa cells, for all DAMP exposure groups, including the mock-exposed control, inclusions also increased in size between 39 hpi (Fig 9 left) and 24 hours later despite continued DAMP exposure (Fig 9 middle). However, as noted for *C*. *pecorum* (Fig 8), the density of bacteria within the *C*. *trachomatis* inclusions, reduced by the initial 39-hour exposure to cAMP, ATP or EHNA/ADO (Fig 9 left), did not substantially increase upon 24 hours of continued DAMP exposure (Fig 9 middle). In contrast, in *C*. *trachomatis-*infected cells exposed to cAMP, ATP or EHNA/ADO for 39 hours then allowed to recover in the absence of DAMP exposure for 24 hours (Fig 9 right), both the size and visual bacterial density of inclusions increased substantially compared to similar infected cells subjected to continued DAMP exposure (Fig 9 middle), although EHNA/ADO-exposed infected cells showed a more pronounced recovery of bacterial density within the inclusions than cAMP- or ATP-exposed cells.

In both *C*. *pecorum*- and *C*. *trachomatis*-infected cells, for all experimental groups some cell loss was seen upon 24 hours of continued incubation, regardless of continued DAMP exposure or recovery conditions. However, in all cases, cell loss was greater under recovery conditions than under continued DAMP exposure conditions (Figs 8 and 9), suggesting that continued infection, not continued exposure, is responsible for the observed cell loss. Because the cells and inclusions remaining may or may not accurately represent inclusion size for an entire coverslip, quantitative measurements were not carried out in these experiments. Thus, results presented are based on qualitative observations of approximately 20 1000x oil-immersion fields per coverslip and images are representative of similar results observed in two independent experiments.

To evaluate the impact of continued DAMP exposure or recovery conditions on subsequent infectious EB production in DAMP-exposed, *Chlamydia*-infected cells, in light of the observed differential cell loss between mock-exposed and DAMP-exposed groups, cells and culture supernatants were collected after 35- or 39-hour exposure to DAMPs followed by 24 hours of continued exposure or recovery. To accurately represent total infectious EB, both released into the supernatant and retained within intact inclusions and cells, the collected cell culture supernatant was combined with collected cells for each sample. These samples were subjected to freeze/thaw and sonication to release EB, used to re-infect HeLa cells, and IFU/ml was determined (7 C and D).

As expected, based upon the microscopically observed increases in inclusion size and bacterial density upon recovery from cAMP and ATP exposure of *C*. *pecorum*-infected cells (Fig 8F and 8I) compared to *C*. *pecorum*-infected cells continuously exposed to these DAMPs (Fig 8E and 8H), IFU/ml was increased in these recovery groups, compared to the correlating continuously exposed groups (Fig 7C). Additionally, EHNA/ADO-exposed *C*. *pecorum*-infected cells also showed an increase in IFU/ml in recovery versus continuous exposure groups (Fig 7C), suggesting that the increased cell loss observed microscopically for the EHNA/ADO recovery group (Fig 8L) compared to the continuously exposed group (Fig 8K) was due to relatively more robust chlamydial growth.

In contrast to the results seen for *C*. *pecorum* (Fig 8), although *C*. *trachomatis* inclusions showed comparatively increased size and visible bacterial density upon recovery from cAMP, ATP, and EHNA/ADO exposure (Fig 9), a concomitant increase in IFU/ml was seen only in the EHNA/ADO-exposed group (Fig 7D). This suggests that the inclusions in cAMP- and ATP- exposed groups (Fig 9) may not produce infectious EB after 24 hours of recovery, despite a relatively normal IF microscopic appearance. This is also in agreement with the lack of close correlation between reduced inclusion size (Fig 3C and 3D) and reduced IFU/ml (Fig 7A and 7B) previously discussed.

### cAMP, ATP, and EHNA/ADO similarly, though less dramatically, reduce inclusion size when *Chlamydia*-infected cells are exposed at 14 hpi versus 0 hpi

**Fig 10:**
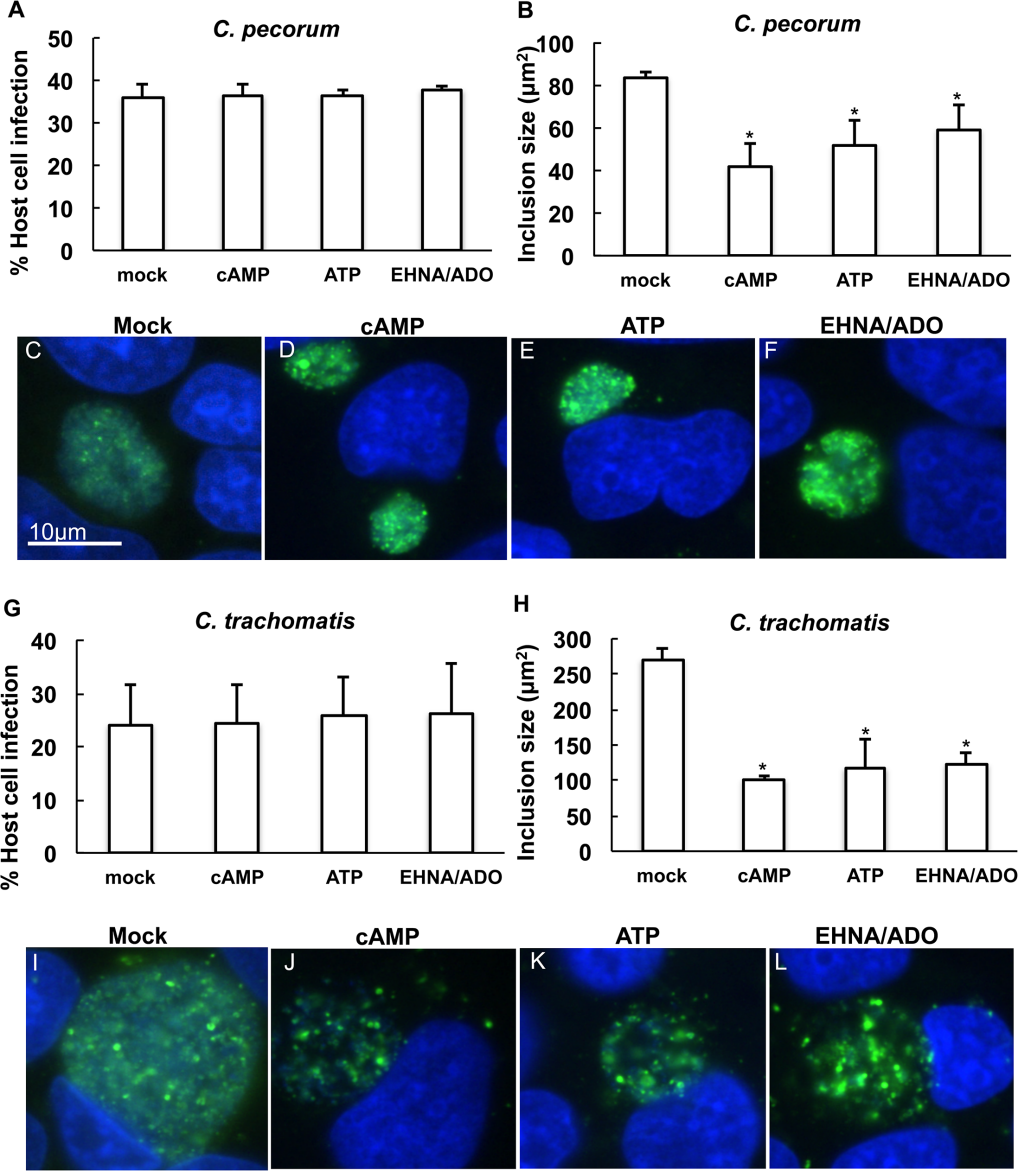
cAMP, ATP and EHNA/ADO inhibit inclusion development less dramatically when added at 14 hpi. HeLa cells were infected with *C*. *pecorum* or *C*. *trachomatis* serovar E and exposed to 1 mM cAMP, 1 mM ATP, or 50 μM ADO plus 25 μM EHNA in incubation medium 14 hours after infection. Cells were fixed and labeled with anti-LPS (green) and DAPI (blue) at 35 hours post infection (*C*. *pecorum*) or 39 hours post infection (*C*. *trachomatis*). Number of inclusions per nucleus was determined and percent infection was calculated for *C*. *pecorum* (A) and *C*. *trachomatis* (G) (mean ± SD; p > 0.05 in all cases, *t* test; n = 3). Mean inclusion size was determined for *C*. *pecorum* (B) and *C*. *trachomatis* (H) (mean ± SD; *p ≤ 0.05, *t* test; n = 3). Representative images are shown for *C*. *pecorum*-infected mock- (C), cAMP- (D), ATP- (E), and EHNA/ADO-exposed (F) cells and for *C*. *trachomatis*-infected mock- (I), cAMP- (J), ATP- (K), and EHNA/ADO-exposed (L) cells. Immunofluorescence microscopy images represent one experiment of three independently repeated experiments with similar results.

Previously, we have shown that PEDV reversibly reduces productive development of both *C*. *pecorum* and *C*. *abortus* and results in conspicuous AB formation, features consistent with chlamydial persistence/stress [6]. Because PEDV-dependent induction of these effects occurred upon exposure of *Chlamydia*-infected host cells to PEDV at 14 hours post chlamydial infection, we postulated whether the timing of exposure to the persistence inducer might exert an influence on the microscopic appearance of the chlamydial inclusions, with regard to AB formation. Thus we evaluated the effect exposure of *Chlamydia*-infected cells to DAMPs at 14 hpi (Figs 1B and 10).

Exposure of *C*. *pecorum*-infected (Fig 10A) and *C*. *trachomatis*-infected (Fig 10G) cells to DAMPs at 14 hpi had no effect on percent of host cells infected. Inclusion size was reduced by 14 hpi DAMP exposure of *C*. *pecorum*-infected (Fig 10B) and *C*. *trachomatis*-infected (Fig 10H) cells, although the degree of this reduction was less pronounced than that observed for such exposure immediately post infection (Fig 3C and 3D). *C*. *pecorum* mean inclusion size was reduced to 49.8% of control with the addition of cAMP, 61.5% of control with the addition of ATP, and 70.9% of control with EHNA/ADO at 14 hpi (Fig 10B). *C*. *trachomatis* mean inclusion size was reduced to 37.5% of control with the addition of cAMP, 43.4% of control with the addition of ATP, and 45.6% of control with EHNA/ADO at 14 hpi (Fig 10H).

The appearance, by IF microscopy, of *C*. *pecorum* (Fig 10C–10F) and *C*. *trachomatis* (Fig 10I–10L) in cells treated with DAMPs at 14 hpi was similar to that seen upon exposure of infected cells immediately post infection (Figs 4 and 5), and, as for earlier exposure no increase in the presence of AB within the inclusions was noted. As observed in similar exposure of *Chlamydia*-infected host cells with DAMPs added immediately after infection, exposure at 14 hpi did not result in host cell loss from the monolayer (S3H and S3I Fig).

## Discussion

In summary, the present study reports that DAMPs, including extracellular ATP and adenosine, and cyclic AMP, which posses extracellular signaling capabilities, inhibit *Chlamydia pecorum* inclusion development *in vitro* in the absence of *de novo* host cell protein synthesis. Under host protein synthesis limitations, each of these DAMPs induce similar alterations of the *C*. *pecorum* developmental cycle and, in the case of adenosine, of the *C*. *trachomatis* serotype E developmental cycle, to those previously reported for *C*. *caviae*, *C*. *muridarum* and *C*. *trachomatis* serotypes D and L2 [11,12,14,27]. These changes include (i) reduction in inclusion size and bacterial density within inclusions, without AB formation; (ii) reduction in release of infectious EB; and (iii) recovery of infectious EB production upon removal of DAMP stimulus. The latter two changes are consistent with the currently accepted definition of persistence or the chlamydial stress response, namely viable, but non-infectious chlamydiae; while lack of aberrant body formation is atypical in chlamydial persistence, but not entirely unrepresented [26]. For example, iron deprivation reduced *C*. *trachomatis* inclusion size without inducing AB formation, though RB morphology was affected, exhibiting wavy cell membranes on the normal-sized RB (determined by electron microscopic analysis) [33]. While in interferon-dependent persistence/chlamydial stress induction, host cell species determines a difference in AB formation, with AB formation associated with human host cell lines [26,34] but not with murine cell lines [34,35]. Notably, *C*. *trachomatis* serotype E failed to recover infectious EB production within 24 hours after discontinuation of cAMP or ATP exposure, an effect which can be explained as killing of the chlamydiae, rather than persistence induction. Further evaluation of possible recovery over extended times may reveal whether *C*. *trachomatis* serotype E is incapable of recovery from cAMP or ATP exposure under certain experimental conditions, or whether species-specific differences exist in the timing of recovery of chlamydiae from DAMPs.

Importantly, the evaluation of inhibition of inclusion development and subsequent recovery from DAMPs reported here was evaluated in the presence of cycloheximide, and thus conclusions about species-specific differences in inhibition by DAMPs and recovery from DAMPs must be made in the context of significantly reduced or abolished host cell *de novo* protein synthesis. In fact, as determined specifically by parallel evaluation of DAMPs exposure in the presence or absence of cycloheximide, in the absence of cycloheximide, cAMP and ATP exposure elicited a markedly different effect on infectious EB production, compared to exposure in the presence of cycloheximide, in both species of chlamydiae tested. In the presence of cycloheximide, both species showed dramatic cAMP- and ATP-dependent reductions in infectious EB production. However, in the absence of cycloheximide, this effect was reversed for *C*. *pecorum*, which showed cAMP- and ATP-dependent increases in infectious EB production. Likewise, the effect was reversed for *C*. *trachomatis* upon ATP exposure, and reduced to statistical similarity with the control upon cAMP exposure. EHNA/ADO exposure, however, elicited a reduction in IFU/ml compared to the control both in the presence or absence of cycloheximide for both chlamydial species evaluated, though a more marked reduction was observed in the presence of cycloheximide for both species. Interestingly, the reductive effect on inclusion size observed for all DAMPs and both chlamydial species in the presence of cycloheximide was also observed in the absence of cycloheximide, though, again, to a lesser extent.

The fluorescence and electron microscopic analysis reported here confirms that viable but non-infectious chlamydiae may be induced upon exposure to DAMPs in culture, but such stressed RB need not be enlarged and irregularly shaped to exhibit the observed reversible reduction in chlamydial infectivity. In fact, the conspicuous reduction in bacterial numbers, with no obvious alterations to EB or RB microscopic appearance suggests that DAMP-induced chlamydial biological changes, though currently unknown, markedly differ from reported changes associated with other persistence inducers such as antibiotic exposure, cytokine exposure, or viral co-infection. For example, smaller-sized inclusions, populated exclusively with grossly enlarged AB, were previously seen in PEDV-induced aberrant *C*. *pecorum* inclusion morphology [6]. In the current study, we evaluated the effect on inclusion formation when cAMP, ATP, or EHNA/ADO were added at 14 hpi, specifically to address the possibility that the time after chlamydial infection of exposure to an inducer of persistence/chlamydial stress might influence AB production. However, DAMPs added at 14 hpi, compared to 0 hpi, also failed to induce AB formation, and instead exerted an effect on chlamydial inclusion development equivalent, if somewhat less dramatic, to that seen at 0 hpi exposure. This indicates that the inducer of aberrant chlamydial inclusions, not the time of induction, is responsible for the disparate appearance of aberrant inclusions in *Chlamydia*-infected cells treated with cAMP, ATP, or EHNA/ADO versus those exposed to PEDV. Furthermore, this suggests that while release of DAMPs from PEDV-infected cells may influence the *C*. *pecorum* developmental cycle, the AB formation and persistence/chlamydial stress observed in the previously reported *C*. *pecorum*/PEDV co-infection model is not likely to be entirely ATP-, cAMP-, or EHNA/ADO-dependent.

One may speculate that the apparent bacterial morphology difference in DAMP-induced persistence/chlamydial stress represents a fundamentally different mode of action than that effecting AB-associated persistence/chlamydial stress. The phenomenon of interferon-induced persistence/chlamydial stress has been well studied, and it is known that interferon elicits its effects on chlamydiae dissimilarly in human versus murine host cells. In human cells the primary effector of persistence is indoleamine dioxygenase (IDO), the activation of which depletes host intracellular tryptophan levels needed for chlamydial growth; while in murine cells, persistence is induced in the absence of IDO activation and can involve nitric oxide synthase (NOs) activation, lipid acquisition disruption, or increased fusion of inclusions with lysosomes [34,36]. The mechanisms for virus co-infection-induced persistence/chlamydial stress was demonstrated to be imbalanced oxidative stress in the case of human herpes virus 6 [5]. In contrast, herpes simplex virus type-2 mediated down-regulation of host nectin-1 protein appears to induce *C*. *trachomatis* persistence [37]. The involvement of these modes of action has not yet been investigated in the context of DAMP-induced persistence/chlamydial stress, and currently, evaluation of possible modes of action for DAMP-induced persistence/chlamydial stress has been limited.

In a study in which cAMP was shown to reversibly reduce chlamydial inclusion size and infectivity, reduction in EB-specific antigens was noted in cAMP-exposed *C*. *trachomatis*, suggesting to the authors that cAMP exerted its effect on *C*. *trachomatis* by arresting development at the RB stage, though no mode of action was proposed [24]. In an earlier study that reported inhibition of *C*. *trachomatis* infectivity in HeLa cells exposed to cell-permeant cAMP before infection, pretreatment with an ionophore which selectively increases the level of intracellular calcium abolished the anti-chlamydial effect of cAMP. However, a reduction in intracellular calcium was not demonstrated upon cAMP exposure, though an increase in calcium mobilization was noted upon chlamydial infection alone [25].

The effects of extracellular ATP on chlamydial persistence/stress in various cell types has been demonstrated, in a series of several studies, to act via the P2X_7_ receptor both *in vitro* and in a murine infection model, and under some conditions the P2X_4_ receptor was also shown to play a role in persistence induction [11–13,27]. In both macrophages and HeLa cells, ATP signaling via the P2X_7_ receptor was demonstrated to act at least in part via phospholipase D activation; in the case of macrophages this was associated with lysosome/vacuole fusion [12,13]. Finally, extracellular adenosine-dependent induction of chlamydial persistence/stress was shown to act via A2b receptor stimulation, and although this was demonstrated to be cAMP-dependent protein kinase (PKA)-independent, the authors noted that both cell-permeant and non-permeant cAMP exerted a similar effect as adenosine and postulated that cAMP is downstream of adenosine signaling and might be responsible for the observed effects [14]. Notably, adenosine-dependent induction of chlamydial persistence/stress was reported to occur in both the presence and absence of cycloheximide, leading the authors to conclude that *de novo* host cell protein synthesis was not required for the observed effects of adenosine [14]. Likewise, the data reported here, in which cycloheximide was included in the incubation medium and remained on the cells for the duration of the experiments, further supports the previous findings and conclusion. Furthermore, we can conclude from parallel evaluation of the effects of DAMPs in the presence/absence of cycloheximide, that extracellular adenosine can exert similar anti-chlamydial effects in the absence or presence of new host cell protein synthesis, while anti-chlamydial effects of cAMP and ATP are abolished, or even reversed, when host cells have uninhibited protein synthesis capabilities.

The marked DAMP-dependent reductions in inclusion size and infectious EB production demonstrated for both *C*. *pecorum* and *C*. *trachomatis*, were accompanied by changes in the relative proportion of the different chlamydial developmental forms within TEM-observed inclusions. Interestingly, the specific changes effected in these relative proportions were starkly different in the two chlamydial species. *C*. *pecorum* exhibited an increase in the proportion of RB, and a concomitant decrease in the proportion of EB, upon exposure to cAMP or ATP. In contrast, *C*. *trachomatis* exhibited the opposite effect: a decrease in the proportion of RB, and a concomitant increase in the proportion of EB, upon cAMP or ATP exposure. This might offer an explanation for the ability of *C*. *pecorum*, but not *C*. *trachomatis*, to recover from cAMP or ATP exposure under the experimental conditions evaluated. Notably dissimilar to cAMP and ATP, EHNA/ADO did not effect changes in the relative proportions of RB and EB for either chlamydial species.

Though the current study does not specifically address possible mechanisms of persistence induction, we do not expect that the pronounced effect of DAMPs on chlamydial inclusion development is due to marked host cell death or damage, since microscopically (IF and TEM) host cells appeared unaltered by any DAMP exposure up to 39 hours post chlamydial infection. In support of this, the exposure of *C*. *trachomatis* L2-infected HeLa cells to 1 mM cAMP has previously been reported to inhibit normal inclusion development, with no indicated observation of cytotoxicity or cell loss upon 48 hours of treatment [24]. Additionally, the addition of 25 μM EHNA/ 50 μM ADO to *C*. *trachomatis* L2- and *C*. *trachomatis* D-infected HeLa cells was previously shown to inhibit chlamydial inclusion development, and cytotoxic effects were not reported [14]. In contrast, extracellular ATP, at concentrations including 1 mM, is well known to induce apoptosis in various cell types, particularly immune cells and transformed cells, and causes a characteristic microscopic appearance including reduced cell surface microvilli, shrunken cells with increased membrane blebbing, and nuclear condensation and/or fragmentation [28,29]. However, we observed no signs of ATP-induced cytotoxicity at any concentration evaluated (by fluorescence or electron microscopy). In support of this finding, (i) previous studies have indicated cycloheximide (used at 1 μg/ml in incubation medium in all experiments shown here) abrogates the morphological effects of ATP-induced apoptosis [28]; (ii) HeLa cells are resistant to extracellular ATP-induced apoptosis, a characteristic thought to be attributable to expression of high levels of a truncated purinergic receptor (P2X_7_) [13] and/or expression of human papillomavirus oncogenes that may offer additional protection against apoptosis [38]; and, finally, (iii) *Chlamydia*-infected cells, including epithelial cells, are protected against induced apoptosis [39].

Data from this study shows that *C*. *trachomatis*, which exhibits no significant recovery in infectious EB production after cAMP or ATP exposure, appears to be more sensitive to DAMPs than *C*. *pecorum*, which significantly recovered infectious EB production after exposure to these molecules, suggesting that species-specific difference exist in the degree of chlamydial response to DAMPs. Both chlamydial species, however, recovered significantly after exposure to ADO, indicating that all DAMPs evaluated in this study might not be exerting an identical effect on the chlamydiae. Additionally, our data suggest that cAMP can exert a similar effect whether or not the molecule is cell-permeant, however, we cannot rule out some entry into the host cells of non-cell-permeant cAMP, making it unclear whether or not cAMP and 8BrCAMP are acting via a shared or distinct mechanism. Also, we showed that exposure of *C*. *pecorum*-infected cells to apyrase alone, an enzyme that catalyzes the hydrolysis of ATP, added in the absence of addition of exogenous ATP, increased inclusion size compared to mock treated controls, though this reduction did not reach statistical significance. This suggests that apyrase reduces levels of endogenous ATP, which thus may be expected to limit chlamydial development during the course of infection, at least *in vitro*.

We also report that infectious EB production in DAMP-exposed chlamydiae does not in all cases correlate well with inclusion size or mean bacteria per inclusion. For example, in the presence of cycloheximide, *C*. *pecorum* showed a greater than 99% decrease in the production of infectious EB upon exposure to EHNA/ADO, despite the fact that corresponding mean bacteria per inclusion was reduced by only 36%, suggesting that microscopically typical-appearing bacteria were in fact non-infectious after exposure to DAMPs. Because, in the absence of cycloheximide, cAMP- and ATP-dependent reduced chlamydial inclusion size still failed to correlate with reduced infectious EB production, we can conclude that, both in the presence and absence of *de novo* host protein synthesis, inclusion size is not a good measure of infectious EB production in all cases. This is in agreement with a recent report indicating that chlamydial protein synthesis, but not chlamydial replication, is required for normal expansion of *C*. *trachomatis* inclusions [40]. It can thus be speculated that the DAMP-dependent reduction of inclusion size observed for *C*. *pecorum* and *C*. *trachomatis* serotype E occurs, in part, independently of host cell *de novo* protein synthesis, and may result from mechanisms requiring bacterial protein synthesis. In contrast, the cAMP- and ATP-dependent reduction of infectious EB release by the chlamydial species evaluated appears to depend on inhibited host cell protein synthesis.

Recovery, as expected, appears to depend upon the re-entry of chlamydiae into the normal developmental cycle, since the two groups that did not recover within 24 hours of discontinuation of DAMP-exposure, namely *C*. *trachomatis* exposed to cAMP or ATP, showed a reduction in mean bacteria per inclusion of at least 94%, more in line with the infectious EB reduction. This additionally supports the idea that DAMPs may induce persistence under some circumstances but may cause irreversible inhibition (killing) of chlamydial development under other circumstances. In conclusion, while the mechanisms responsible for DAMP-dependent chlamydial persistence/stress remain to be elucidated, the data presented here, along with that of previous studies evaluating DAMP-induced persistence/chlamydial stress, suggests damage/danger signaling may play a significant role in the host/pathogen interactions that regulate chlamydial infections. *In vivo* concentrations of the evaluated DAMPs vary, but are generally relatively low. Intracellular ATP levels are typically in the 3–10 mM range, with extracellular levels rising into the hundreds of μM range under physiological stress [41]. Adenosine tissue levels are normally in the nM range, but may rise as high as 100 μM in inflamed/ischemic tissues [42]. Normal physiological cAMP levels approach the μM range, however, broad variations in cAMP levels induced by various stimuli in various tissues are of unknown biological importance [43]. Because lysis of host cells during *Chlamydia* infection, and various other infections, has the potential to markedly increase local concentrations of DAMPs at the site of infection, DAMPs may play a role in the progression of Chlamydia infection *in vivo*, particularly in the context of poly-microbial infections.

## Supporting Information

S1 Fig DAMPs reduce inclusion size in the presence or absence of new host cell protein synthesis. HeLa cells were infected with *C*. *pecorum* (A-H, Q-R) or *C*. *trachomatis* serovar E (I-P,S-T) and exposed to the DAMPs cAMP (1 mM), ATP (1 mM), or ADO (50 μM, plus 25 μM EHNA) in incubation medium, in the presence (A-D, I-L, Q, S) or absence (E-H, M-P, R, T) of 1 μg/ml cycloheximide, immediately after infection. Cells were incubated for 35 hours (*C*. *pecorum*) or 39 hours (*C*. *trachomatis*), fixed and labeled with anti-LPS and DAPI, and inclusion size was determined (mean ± SD; * p ≤ 0.0002, *t* test; n = 50 inclusions per coverslip from a single experiment).
(TIFF)

S2 Fig cAMP and ATP modulate the proportion of chlamydial developmental forms within inclusions. HeLa cells were infected with *C*. *pecorum* or *C*. *trachomatis* serovar E and exposed to the DAMPs cAMP (1 mM), ATP (1 mM), or ADO (50 μM, plus 25 μM EHNA) in incubation medium immediately after infection. Cells were incubated for 35 hours (*C*. *pecorum*) or 39 hours (*C*. *trachomatis*), fixed, and processed by standard methods for TEM analysis. Total number of bacteria (EB, RB, IB, and AB) in ten inclusions per condition was counted, the proportion of each bacterial type per inclusion was determined, and mean proportion of developmental forms for the 10 inclusions per experimental group were calculated (mean ± SD; *p = 0.05, *t* test; n = 10 inclusions from a single experiment). The relative proportions of EB, RB, IB and AB are shown for *C*. *pecorum* (A) and *C*. *trachomatis* (B).
(TIFF)

S3 Fig cAMP, ATP and EHNA/ADO do not cause host cell loss. HeLa cells were infected with *C*. *pecorum* or *C*. *trachomatis* serovar E and exposed to cAMP (1 mM), 8BrcAMP (1 mM), ATP (1 mM), Apyrase (2.5 U), Apyrase (2.5 U) followed by ATP (1 mM), ADO (50 μM), EHNA (25 μM), or ADO (50 μM) plus EHNA (25 μM) in incubation medium immediately after infection (T_0_; A-G) or 14 hours post infection (T_14_; H,I). Cells were incubated for 35 hours (*C*. *pecorum*) or 39 hours (*C*. *trachomatis*) from T_0_, then fixed and labeled with anti-LPS and DAPI. Number of nuclei was determined and mean nuclei per field was calculated (mean ± SD; p >0.05 in all cases, *t* test; n = 3 A-D and H-I, n = 8 fields per coverslip from a single experiment E-G).
(TIFF)

S4 Fig DAMP-dependent modulation of infectious EB production depends on host cell *de novo* protein synthesis. HeLa cells were infected with *C*. *pecorum* (A-B) or *C*. *trachomatis* serovar E (C-D) and exposed to the DAMPs cAMP (1 mM), ATP (1 mM), or ADO (50 μM, plus 25 μM EHNA) in incubation medium, in the presence (A, C) or absence (B, D) of 1 μg/ml cycloheximide, immediately after infection. Cells were incubated for 35 hours (*C*. *pecorum*) or 39 hours (*C*. *trachomatis*). Infected monolayers were then collected and processed for titration by sub-passage. Number of inclusions was determined and inclusion forming units (IFU) per ml was calculated (mean ± SD, *p ≤ 0.05, *t* test; values are derived from duplicate determinations within a single experiment).
(TIFF)
