# Supplementary figures and images for: Damage/Danger Associated Molecular Patterns (DAMPs) Modulate Chlamydia pecorum and C. trachomatis Serovar E Inclusion Development In Vitro
Source: PLoS One. 2015 Aug 6;10(8):e0134943. doi: 10.1371/journal.pone.0134943 (PMC4527707; doi:10.1371/journal.pone.0134943)

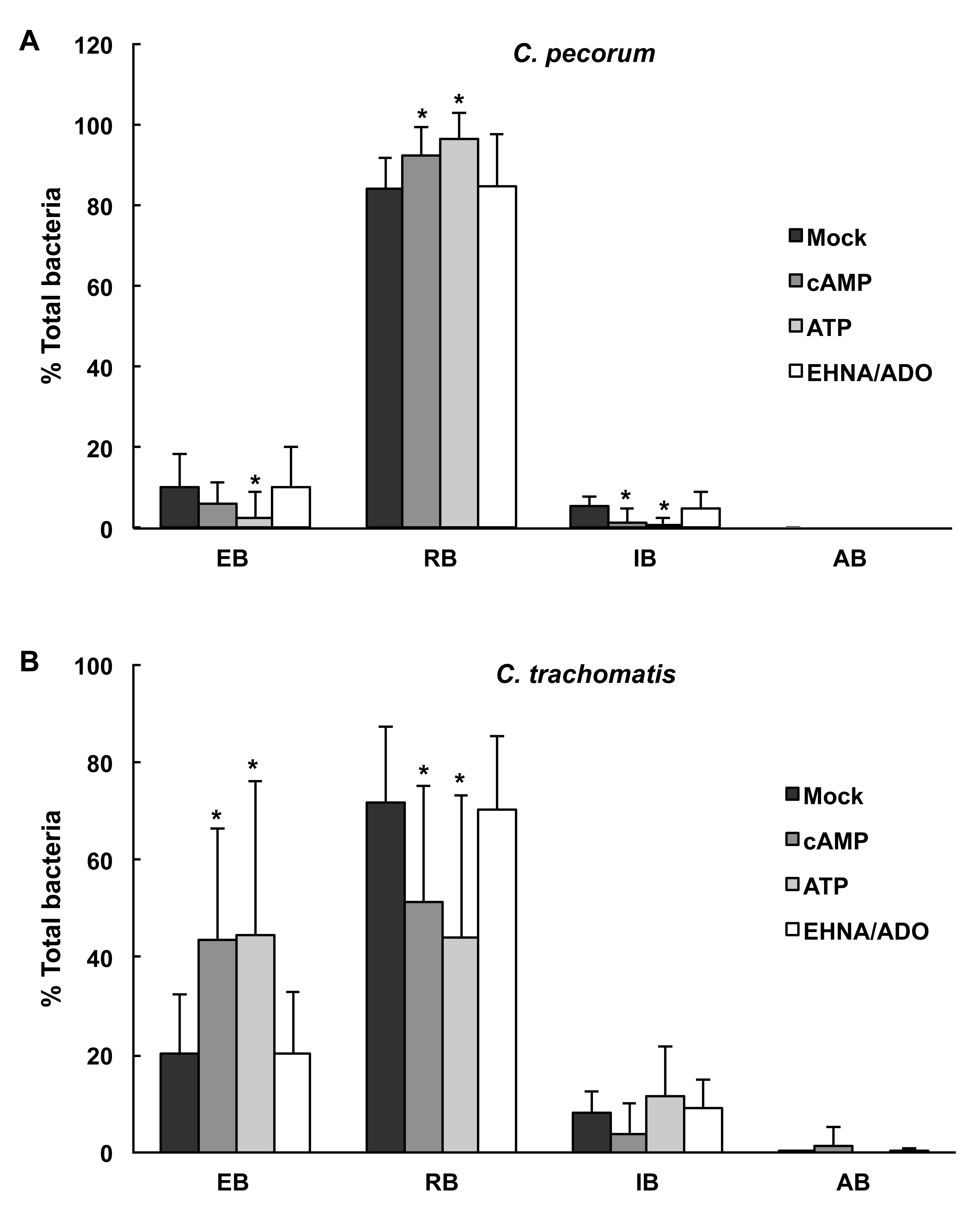

Supplement: S2 Fig — HeLa cells were infected with C. pecorum or C. trachomatis serovar E and exposed to the DAMPs cAMP (1 mM), ATP (1 mM), or ADO (50 μM, plus 25 μM EHNA) in incubation medium immediately after infection. Cells were incubated for 35 hours (C. pecorum) or 39 hours (C. trachomatis), fixed, and processed by standard methods for TEM analysis. Total number of bacteria (EB, RB, IB, and AB) in ten inclusions per condition was counted, the proportion of each bacterial type per inclusion was determined, and mean proportion of developmental forms for the 10 inclusions per experimental group were calculated (mean ± SD; *p = 0.05, t test; n = 10 inclusions from a single experiment). The relative proportions of EB, RB, IB and AB are shown for C. pecorum (A) and C. trachomatis (B). (TIFF) [file pone.0134943.s002.tiff]

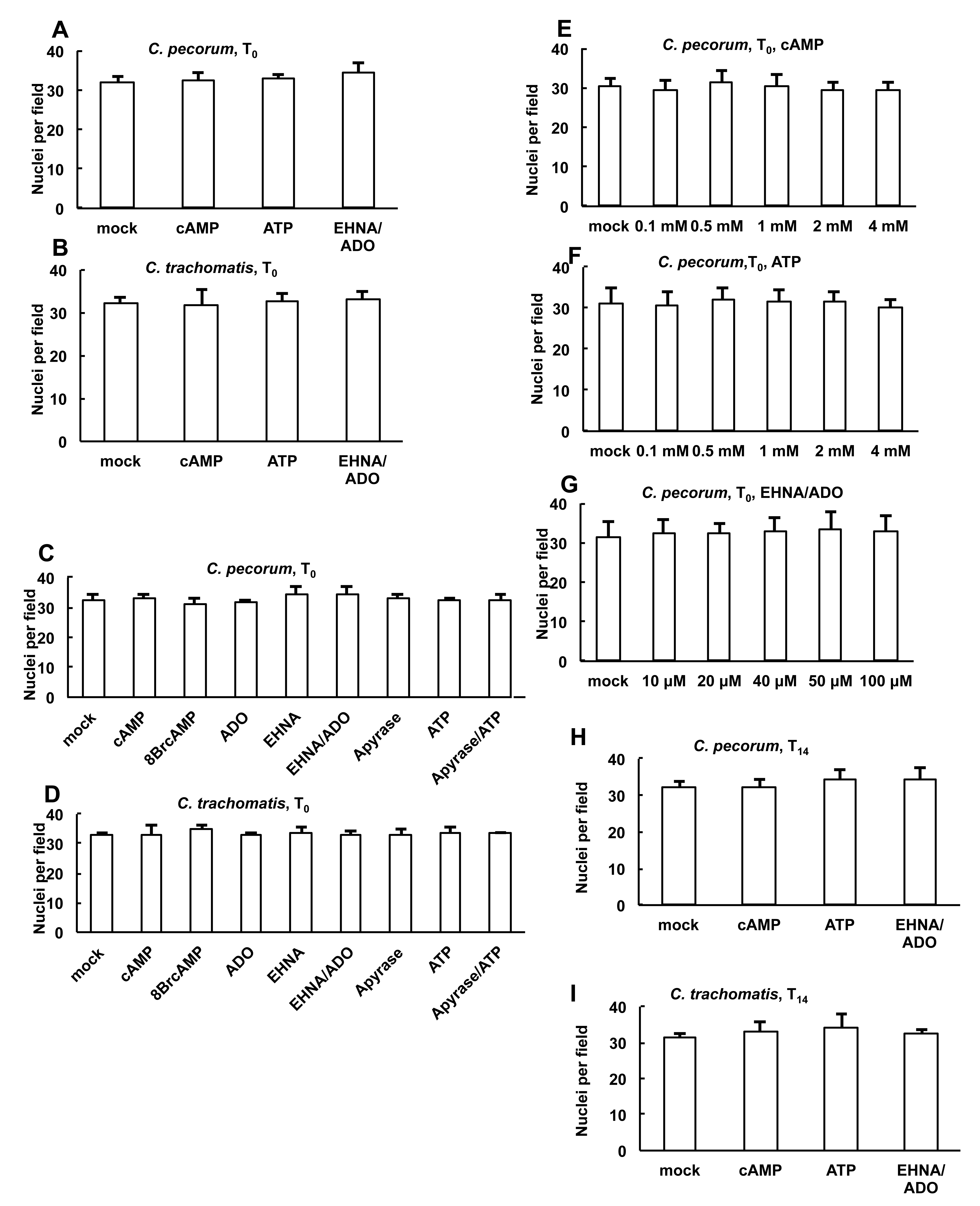

Supplement: S3 Fig — HeLa cells were infected with C. pecorum or C. trachomatis serovar E and exposed to cAMP (1 mM), 8BrcAMP (1 mM), ATP (1 mM), Apyrase (2.5 U), Apyrase (2.5 U) followed by ATP (1 mM), ADO (50 μM), EHNA (25 μM), or ADO (50 μM) plus EHNA (25 μM) in incubation medium immediately after infection (T0; A-G) or 14 hours post infection (T14; H,I). Cells were incubated for 35 hours (C. pecorum) or 39 hours (C. trachomatis) from T0, then fixed and labeled with anti-LPS and DAPI. Number of nuclei was determined and mean nuclei per field was calculated (mean ± SD; p >0.05 in all cases, t test; n = 3 A-D and H-I, n = 8 fields per coverslip from a single experiment E-G). (TIFF) [file pone.0134943.s003.tiff]

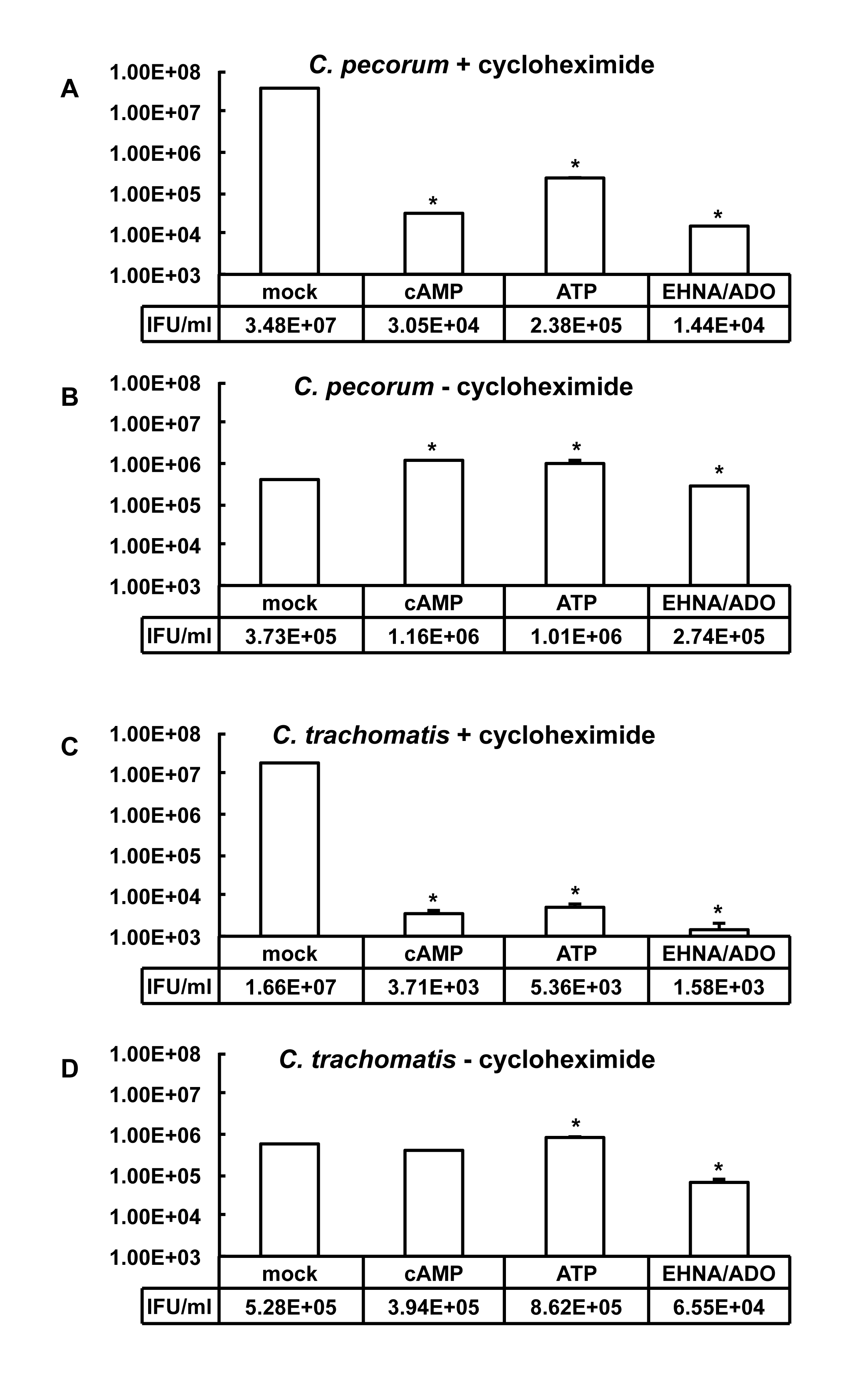

Supplement: S4 Fig — HeLa cells were infected with C. pecorum (A-B) or C. trachomatis serovar E (C-D) and exposed to the DAMPs cAMP (1 mM), ATP (1 mM), or ADO (50 μM, plus 25 μM EHNA) in incubation medium, in the presence (A, C) or absence (B, D) of 1 μg/ml cycloheximide, immediately after infection. Cells were incubated for 35 hours (C. pecorum) or 39 hours (C. trachomatis). Infected monolayers were then collected and processed for titration by sub-passage. Number of inclusions was determined and inclusion forming units (IFU) per ml was calculated (mean ± SD, *p ≤ 0.05, t test; values are derived from duplicate determinations within a single experiment). (TIFF) [file pone.0134943.s004.tiff]
